# Matrix-assisted laser desorption/ionization time-of-flight mass spectrometry traces the geographical source of *Biomphalaria* *pfeifferi* and *Bulinus* *forskalii*, involved in schistosomiasis transmission

**DOI:** 10.1186/s40249-023-01168-y

**Published:** 2024-01-29

**Authors:** Papa Mouhamadou Gaye, El Hadj Ibrahima Ndiaye, Souleymane Doucouré, Doudou Sow, Mapenda Gaye, Ndiaw Goumballa, Carole Cassagne, Coralie L’Ollivier, Oleg Medianikov, Cheikh Sokhna, Stéphane Ranque

**Affiliations:** 1 Aix-Marseille University, IRD, AP-HM, SSA, VITROME, 13005 Marseille, France; 2 VITROME, International IRD-UCAD Campus, 1386 Dakar, Senegal; 3 Hospital-University Institut (IHU) Mediterranée Infection, 13005 Marseille, France; 4 Departement of Animal Biology, Faculty of Sciences and Techniques, UCAD, 5005 Dakar, Senegal; 5 Department of Parasitology-Mycology, UFR Sciences de la Santé, Université Gaston Berger, 234 Saint-Louis, Senegal

**Keywords:** Matrix-assisted laser desorption/ionization time-of-flight mass spectrometry, Snail, Intermediate host, *Biomphalariapfeifferi*, *Bulinusforskalii*, Schistosomiasis, Senegal

## Abstract

**Background:**

Freshwater snails of the genera *Bulinus* spp., *Biomphalaria* spp., and *Oncomelania* spp. are the main intermediate hosts of human and animal schistosomiasis. Identification of these snails has long been based on morphological and/or genomic criteria, which have their limitations. These limitations include a lack of precision for the morphological tool and cost and time for the DNA-based approach. Recently, Matrix-Assisted Laser Desorption/Ionization Time-Of-Flight (MALDI–TOF) mass spectrometry, a new tool used which is routinely in clinical microbiology, has emerged in the field of malacology for the identification of freshwater snails. This study aimed to evaluate the ability of MALDI–TOF MS to identify *Biomphalaria* *pfeifferi* and *Bulinus* *forskalii* snail populations according to their geographical origin.

**Methods:**

This study was conducted on 101 *Bi.* *pfeifferi* and 81 *Bu.* *forskalii* snails collected in three distinct geographical areas of Senegal (the North-East, South-East and central part of the country), and supplemented with wild and laboratory strains. Specimens which had previously been morphologically described were identified by MALDI–TOF MS [identification log score values (LSV) ≥ 1.7], after an initial blind test using the pre-existing database. After DNA-based identification, new reference spectra of *Bi.* *pfeifferi* (*n* = 10) and *Bu.* *forskalii* (*n* = 5) from the geographical areas were added to the MALDI–TOF spectral database. The final blind test against this updated database was performed to assess identification at the geographic source level.

**Results:**

MALDI–TOF MS correctly identified 92.1% of 101 *Bi.* *pfeifferi* snails and 98.8% of 81 *Bu.* *forskalii* snails. At the final blind test, 88% of 166 specimens were correctly identified according to both their species and sampling site, with LSVs ranging from 1.74 to 2.70. The geographical source was adequately identified in 90.1% of 91 *Bi.* *pfeifferi* and 85.3% of 75 *Bu.* *forskalii* samples.

**Conclusions:**

Our findings demonstrate that MALDI–TOF MS can identify and differentiate snail populations according to geographical origin. It outperforms the current DNA-based approaches in discriminating laboratory from wild strains. This inexpensive high-throughput approach is likely to further revolutionise epidemiological studies in areas which are endemic for schistosomiasis.

**Supplementary Information:**

The online version contains supplementary material available at 10.1186/s40249-023-01168-y.

## Background

Schistosomiasis is a neglected tropical disease (NTD) that affects more than 250 million people worldwide [1], including children, young adults, and adults, with an additional 700 million people at risk of infection [2, 3]. It is the second most significant endemic parasitic disease after malaria in terms of its impact on public health [4] with at least 11,792 deaths a year worldwide [5]. The disease is most widespread in low- and middle-income countries in tropical and subtropical areas [1, 2]. In 2021, according to the World Health Organization (WHO), schistosomiasis was mainly confined to sub-Saharan Africa, where an estimated 91% of cases occurred [5]. In this region, prevalence is particularly linked to irrigation works, agricultural activities [6], and poor socio-environmental conditions, including a lack of drinking water, which facilitates contact between humans and water [7]. The disease involves various trematodes of the genus *Schistosoma,* the life cycle of which requires an obligatory passage through a freshwater gastropod snail [8]. Snails of the genera *Biomphalaria* spp., *Bulinus* spp. and *Oncomelania* spp. serve as intermediate hosts for the larval development of these trematodes, the most common of which are *Schistosoma* *haematobium*, *S.* *mansoni* and *S.* *japonicum*, respectively [9, 10]. The transmission of the disease is highly dependent on the expansion of intermediate host snails and the rural development of water resources.

In Senegal, ecological changes following the construction of the Diama and Manantali dams provided new habitats to be colonised by intermediate hosts, resulting in the emergence of human schistosomiasis along the Senegal River [11, 12]. Two clinical types of schistosomiasis are present in Senegal, namely the intestinal form caused by *S.* *mansoni* and the urinary form caused by *S.* *haematobium* [11]. The genus *Biomphalaria* has been confirmed as an intermediate host of *S.* *mansoni* in Africa [13, 14], in particular the species *Bi.* *pfeifferi*, which is found in areas of permanent transmission in Senegal [15, 16]. The urinary form, caused by *S.* *haematobium* and involving snails of the genus *Bulinus*, is found in all parts of Senegal [17]. *Bu.* *senegalensis,* *Bu.* *umbilicatus* and *Bu.* *forskalii* are sympatric species found in the central part of the country, which is characterised by a seasonal transmission of schistosomiasis. We recently reported a 29% prevalence of *S.* *haematobium* complex infection in *Bu.* *senegalensis* and *Bu.* *umbilicatus* snails [7]. *Bu.* *forskalii* is known to be an intermediate host of *Schistosoma* *bovis*, *S.* *intercalatum,* and the *S.* *haematobium*-*intercalatum* hybrid in several countries [18, 19], and we recently showed that *Bu.* *forskalii* also hosts both *S.* *haematobium* and *S.* *haematobium*-*bovis* hybrids in Senegal [20].

Cross-infestations of snails and schistosomes from two different locations in Zimbabwe showed higher compatibility in sympatric strains than in allopatric strains [21]. *S.* *haematobium* from the middle valley of the Senegal River showed some compatibility with *Bu.* *senegalensis* from Matam, while *S.* *haematobium* from the lower valley was incompatible with *Bu.* *senegalensis* [22]. The compatibility between schistosomes and snails seems to be related to the geographical source of the parasite and its intermediate host. It is, therefore, important to be able to identify snails and their parasites according to their respective geographical origins [21, 23]. At the genus level, the species of the genus *Biomphalaria* are morphologically distinct from the *Bulinidae*. However, intraspecific identification in these snails remains complicated and often excludes morphology-based approaches. These morphological methods have many limitations linked to the quality of the specimens, the lack of identification keys or specific documentation and expertise in malacology [24, 25]. These limitations are exacerbated as populations of distinct geographical source are morphologically similar within the same species. Molecular approaches are appropriate but are expensive and limited by the incompleteness of online sequence databases [26].

Matrix-assisted laser desorption/ionization time-of-flight mass spectrometry (MALDI–TOF MS) is a clinical microbiology tool used for the identification of microorganisms [27]. This tool was proposed for arthropod identification in 2005 [28] and then became widely used in entomological studies for the rapid identification of many arthropods, including mosquitoes, ticks, lice, fleas, and bedbugs [29]. Recently, MALDI–TOF MS has been used in malacology to identify and classify edible bivalve snails [30] and medically important gastropods, including species in the *Bulinus* and *Biomphalaria* genera, and other species of Viviparidae [31]. This study aimed to assess the capability of MALDI–TOF MS to identify the geographical source of *Bi.* *pfeifferi* and *Bu.* *forskalii* snails.

## Methods

### Study area

**Fig. 1:**
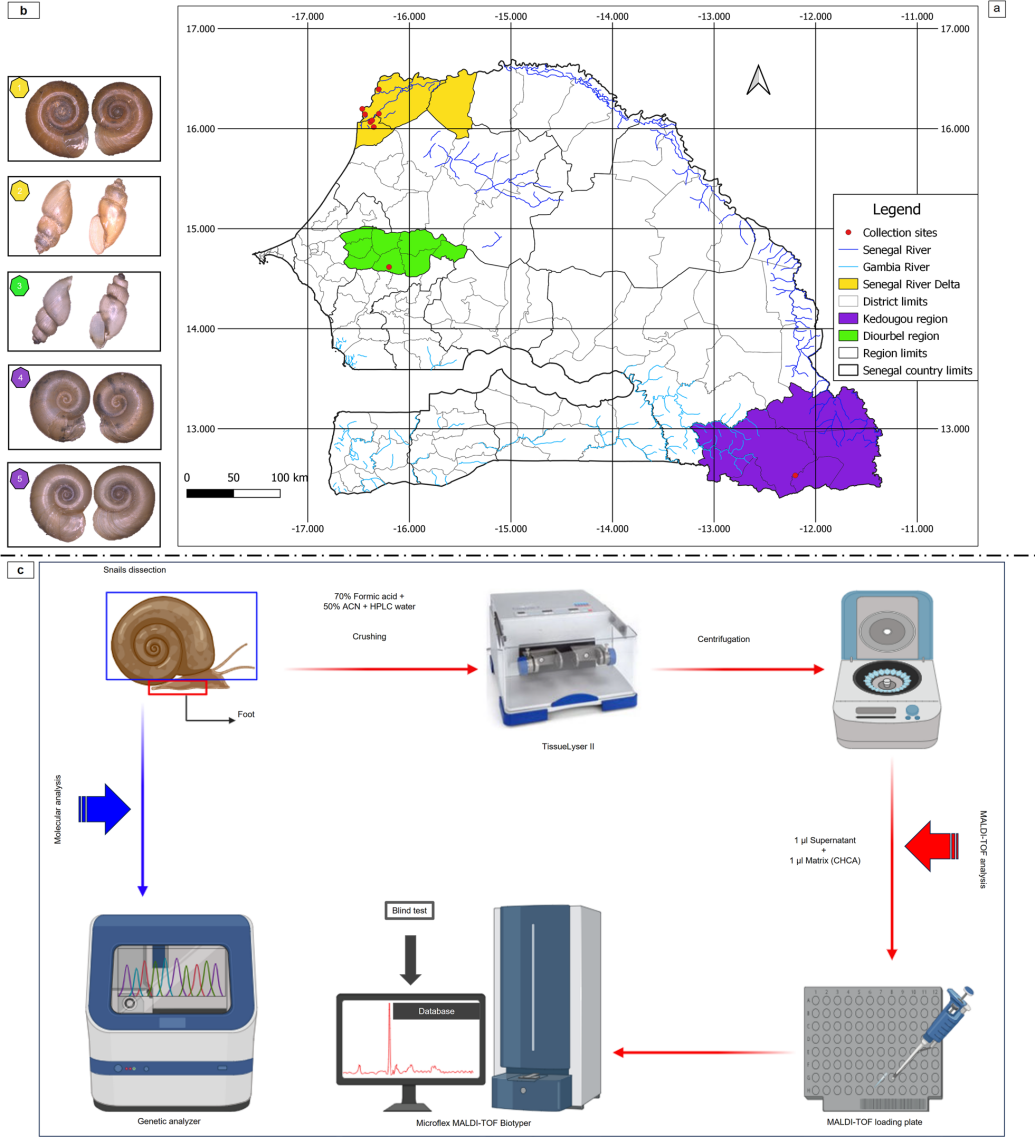
**a** The map of Senegal showing the snail sampling sites, produced using the Geographic Information System software QGIS v3.18.3-Zürich: http://www.qgis.org. **b** The different snail species collected were (1) *Bi.* *pfeifferi* SRD (NW), (2) *Bu.* *forskalii* SRD (NW), (3) *Bu.* *forskalii* Diourbel (CS), (4) *Bi.* *pfeifferi* Kedougou (SE) laboratory strain, and (5) *Bi.* *pfeifferi* SE wild strain. **c** An explanatory flowchart of the MALDI–TOF MS protocol. ACN: Acetonitrile, CHCA: α-cyano-4-hydroxycinnamic acid (created using BioRender.com)

Snails were collected during malacological surveys carried out in September 2020, mainly in the Senegal River Delta (SRD) in the North-West (NW) of Senegal. Specimens were collected in September and November in the Central Senegal (CS), in the Diourbel region, and in the Souht-East (SE) of the country, in the Kedougou region, respectively (Fig. 1a) Both the SRD and the SE of the country are endemic areas for schistosomiasis [11]. We also included in our study a *Biomphalaria* strain originating from Kedougou, which had been raised for two years in a laboratory.

The SRD area is the terminal part of the Senegal River and is located in the North-West of the country between latitudes 16° and 14°40′ North, and longitudes 15°30′ and 16°30′ West [11]. The SRD is a coastal area [32] extending from the Dagana region to Saint-Louis, and covering an area of ~ 6000 km^2^ (Fig. 1a). It is characterised by a semi-desert climate [11] with average minimum temperature of 25.1 ℃ and a maximum of 33.2 ℃. In September 2020, the average rainfall was 188 mm (mean relative humidity, 80.9%) [33]. The area became endemic for schistosomiasis after the construction of the Diama and Manantali dams in the 1980s, and is characterised by stable transmission [34]. In the Delta zone, we surveyed seven sites (Savoigne: 16°10′N/16°18′W, Keur Samba: 16°11′N/16°16′W, Kabane: 16°30′N/16°24′W, Ndiawdoune: 16°40′N/16°23′W, Minguene: 16°01′N/16°21′W, Mbakhana: 16°05′N/16°22′W and Ndiol Maure: 16°09′N/16°18′W), where we collected snails (*n* = 16, *n* = 60, *n* = 39, *n* = 14, *n* = 12, *n* = 6, and *n* = 15, respectively).

In South-Eastern Senegal, snails (*n* = 16) were collected in the Kedougou region at a single site in the village of Ngari (12°32′N, 12°11′W) (Fig. 1a). The Kedougou region is crossed by the Gambia River, close to its source in the Fouta Djallon and tributaries such as the Niokolo Koba [35]. It is characterised by a Sudano-Guinean climate with a single rainy season from May to November [36]. Average minimum temperatures of 23.1 ℃ and 20.7 ℃ and maximum temperatures of 33.1 ℃ and 37.5 ℃, with average monthly rainfall of 226.57 mm and 0.0 mm (mean relative humidity of 79.4% and 42.2%) in September and November 2020, respectively, were recorded [33]. The area features natural water sources, which create favourable biotopes for snails, the intermediate hosts of schistosomiasis [37]. The continued transmission of schistosomiasis in this area is due to the stability of the breeding sites, provided in certain places by dense vegetation that limits the action of solar radiation during the hot periods (40 ℃ between May and June). These permanent watering places concentrate all the villages’ water-based activities [37].

In contrast, Central Senegal is characterised by temporary pools of water, associated with a seasonal transmission of urinary schistosomiasis, depending on the rainy season [7, 17]. Our collection site here was located in the Diourbel region, 150 km east of Dakar, between latitudes 14°30′ and 15° North and longitudes 15°40′ and 16°40′ West [38] (Fig. 1a). It is a semi-urban area, currently not described as endemic for schistosomiasis, but featuring temporary pools. The climate in this area is Sudano-Sahelian, characterised by relatively high temperatures, a long dry season (November to June), and a four-month rainy season (July to October). In September 2020, the average minimum temperature was 24.8 ℃, the maximum temperature was 33.6 ℃ and there was an average monthly rainfall of 419.36 mm (mean relative humidity 82%) [33]. One site in the village of Touba-Ndiareme (14°37′N/16°12′W) was surveyed and four snails were collected.

### Snail collection and morphological identification

With regards to collecting snails from ponds, flexible forceps were used to collect snails from the vegetation surrounding the waterholes or from any other material, including branches and dead leaves. In the river, we scraped the vegetation with a long-handled landing net. The vegetation was then shaken into a container, where the snails fell and were collected. Snails from the same water point were identified morphologically and kept in the same pre-labelled container, noting the collection site, in order to facilitate their transportation. The identification of the snails was based on shell morphology [39]. Some specimens, which were difficult to identify with the naked eye, were observed using a binocular magnifying glass and the Zeiss Axio Zoom V16 microscope (Zeiss, Marly-le-Roi, France) (Fig. 1b). The snails were classified according to species and geographical area, and were then stored at –80 ℃.

### Sample preparation for MALDI–TOF MS analysis

Each snail specimen was carefully extracted from its shell and dissected with a new sterile blade to collect the foot. Each foot was successively rinsed with 70% ethanol and distilled water for two minutes and dried on sterile filter paper for MALDI–TOF MS analysis, while the remainder of the body was stored at −20 ℃ for further genomic analysis. The snail foot was selected because it provides a better source of protein than other tissues for MALDI–TOF MS identification [31, 40]. The feet of the *Bi.* *pfeifferi* and *Bu.* *forskalii* snails were placed individually in 1.5 ml Eppendorf tubes with glass beads, ≤ 106 μm (Sigma, Lyon, France), and mix containing 70% (v/v) formic acid (Sigma-Aldrich, Lyon, France), 50% (v/v) acetonitrile (Fluka, Buchs, Switzerland) and high-quality liquid chromatography (HPLC) water. All samples were ground with 30 μl of the mixture using a TissueLyser II (Qiagen, Hilden, Germany) over three one-minute cycles at 30 Hz.

### Sample loading on the target plate and MALDI–TOF MS settings

The samples were then centrifuged at 2000×*g* for 30 s and 1 μl of the supernatant of each homogenate was deposited on a MALDI–TOF MS target plate (Bruker Daltonics, Wissembourg, France) in ten copies. Each deposit was covered with one microlitre of a CHCA matrix suspension, composed of saturated α-cyano-4-hydroxycynnamic acid (Sigma, Lyon. France), 50% acetonitrile (v/v), 2.5% trifluoroacetic acid (v/v) (Aldrich, Dorset, United Kingdom), and high-performance liquid chromatography (HPLC) water to allow for co-crystallisation. After drying for several minutes at room temperature, the target was introduced into the Microflex LT instrument (Bruker Daltonics, Bremen, Germany) for analysis (Fig. 1c).

### MALDI–TOF spectral analysis

Protein mass profiles were obtained using a Microflex LT instrument (Bruker Daltonics, Germany), using Flex Control version 2.4 software (Bruker Daltonics). This performs positive ion measurements in linear mode at a laser frequency of 50 Hz, in a mass range of 2 kDa to 20 kDa. Mass spectra were analysed over an m/z range of 2000 to 20,000. Each spectrum corresponds to the ions obtained from 240 laser shots fired in six regions of the same deposit on the ground plate and acquired automatically using the AutoXecute function of the FlexControl v.2.4 software (Bruker Daltonics GmbH & Co. KG, Bremen, Germany). Spectral profiles obtained from *Bi.* *pfeifferi* and *Bu.* *forskalii* snail feet were displayed with FlexAnalysis v.3.3 software and exported to ClinProTools v.2.2 (Bruker Daltonics) and MALDI Biotyper v.3.0 (Bruker Daltonics, Germany) for data processing.

Intraspecific reproducibility and interspecific specificity were assessed by comparing the spectral profiles obtained from the ten spots of each snail specimen. Spectral quality was confirmed using FlexAnalysis software making it possible to assess the intensity, peak regularity, baseline flatness, and inter- and intra-group reproducibility of the snails. The original spectra of each snail species were imported into ClinProTools for principal component analysis (PCA). Poor quality spectra (low peak intensity < 3000 arbitrary units (au), and/or no reproducibility) were excluded from the analysis. A dendrogram was created using MALDI Biotyper software to visualise the heterogeneity level of MS spectra from snail groups.

### Blind tests

To confirm the morphological identification of the snails, the MALDI–TOF MS spectra obtained from the foot of each specimen were queried using MALDI Biotyper against our in-house reference spectra database including 64 spectra from eight snail species collected in two areas of Senegal, namely Richard-Toll in the north, and Niakhar in the centre. The level of similarity is estimated by the log score value (LSV) that correspond to the degree of homology between the query and the reference spectra in the database. The blind test report score can range from 0 to 3 depending on the degree of matching between the spectral signal intensities. A sample was considered to be correctly identified when the spectrum analysed provided an LSV value ≥ 1.7. For each sample, four spectra with the highest LSV of the ten were selected for further analysis. The in-lab database contains reference spectra of a number of freshwater gastropod species and is available online at https://doi.org/10.35088/f605-3922: raw-data-frozen-and-ethanol-stored-snails.

### MS data analysis and interpretation

The ClinProTools and MALDI Biotyper software packages (Bruker Daltonics GmbH & Co. KG, Bremen, Germany) were used for data analysis and interpretation. The Flex Analysis software was used to visualise the raw spectra obtained using the MALDI–TOF Microflex LT mass spectrometer. The raw spectral data were processed by PCA to visualise the inter-species MS profile dissimilarities produced by ClinProTools. The same software was used to identify the discriminating peaks between specimens in each of the two species *Bu.* *forskalii* and *Bi.* *pfeifferi*, based on their geographical origin. A dendrogram was also drawn with MALDI Biotyper v.3.0.

The spectra of *Bi.* *pfeifferi* snails collected in the populations located in North-Western Senegal (in the SRD), South-Eastern Senegal (in Kedougou), including both field (WS) and laboratory strains (LS), as well as those of *Bu.* *forskalii* collected in North-Western and Central Senegal (Diourbel), were analysed using ClinProTools. In our analyses, we selected four spectra for each specimen in each geographical zone. For the PCA, all *Bi.* *pfeifferi* specimens collected from the field in Kedougou (WS) (*n* = 6) (MP5Bi1, MP5Bi2, MP5Bi4, MP5Bi5, MP5Bi6 and MP5Bi7) and from the laboratory (LS) (*n* = 10) (KgBi1, KgBi12, KgBi13, KgBi14, KgBi15, KgBi16, KgBi17, KgBi18, KgBi19 and KgBi20) were included. To balance the number of specimens of each geographical origin, we randomly selected ten snails collected in the SRD area for each species (*Bi.* *pfeifferi*: KABiI2, KABiI3, KABiI4, KABiI6, KABiI7, KABi1, KABi5, KABi7, KABi8 and KABi9, and *Bu.* *forskalii*: KABfI1, KABfI2, KABfI3, KABfI4, KABfI5, KABf1, KABf2, KABf3, KABf5 and KABf7). In the centre, in the Diourbel area, we used three specimens of *Bu.* *forskalii* (KhBf1, KhBf2 and KhBf3).

### MALDI–TOF MS biomarker mass set

To determine differential peaks, spectral mass profiles were loaded into ClinProTools (Bruker Daltonics) based on a subset of three *Bu.* *forskalii* samples and twelve *Bi.* *pfeifferi* samples. The parameter settings in ClinProTools for spectral data preparation consisted in baseline subtraction (top-hat; minimum baseline width of 10%), recalibration (maximum peak shift of 1000 ppm and 30% match to calibrant peaks, and exclusion of impossible-to-recalibrate spectra), calculation of the average spectrum (resolution 800), calculation of the average peak list (signal-to-noise threshold 2.00), calculation of peaks in individual spectra, and normalisation of peak lists.

Groups were created for the different snail populations, including group A (population 1 = *Bu.* *forskalii* NW samples and population 2 = *Bu.* *forskalii* Central Senegal samples) and group B (population 3 = *Bi.* *pfeifferi* NW samples and population 4 = *Bi.* *pfeifferi* SE lab strain and wild strain samples).

Biomarker peaks were identified using the “Peak Statistic” function of ClinProTools, followed by manual confirmation that the same peaks could be distinguished using FlexAnalysis. A genetic algorithm in ClinProTools provided the highest “recognition capability” (RC) and “cross-validation” (CV) values with the lowest number of peaks. These values indicate the capacity to split between different classes of spectra based on the selected discriminating peaks.

### DNA extraction and nucleotide sequence analysis

Samples which were well identified, both morphologically and by MALDI–TOF MS (LSV ≥ 1.7), were also randomly selected for further DNA-based identification. For each snail, the remaining specimen was rinsed with distilled water and placed in a 1.5 ml Eppendorf tube for genomic DNA extraction. In short, each sample was incubated at 56 ℃ overnight with 180 μl of G2 lysis buffer (Qiagen Hilden, Germany) and 20 μl of proteinase K (Qiagen Hilden, Germany). The supernatant was recovered in another tube and then extracted using the EZ1 BioRobot extraction device (Qiagen Hilden, Germany) employing the EZ1 DNA Tissue Kit (Qiagen) according to the manufacturer’s instructions. Genomic DNA of each sample was eluted with 200 μl of Tris–EDTA buffer (Qiagen) and stored at – 20 ℃ until use.

For DNA-based identification, the PCR template was DNA extracted from a specimen previously identified by MALDI–TOF MS. PCR reaction targeting a 710 bp region of cytochrome c oxidase subunit I (COI) and a 550 bp region of the 16S rRNA was performed in a thermal cycler (Applied Biosystems, 2720, Foster City, USA) with AmpliTaq Gold 360 PCR master mix (Applied Biosystems, Waltham, USA). The COI region was amplified using Folmer’s universal primers LCO1490 (5′-GGTCAACAAATCATAAAGAT ATTGG-3′), HCO2198 (5′-TAAACTTCAGGGTGACCAAAAAATCA-3′) [41, 42] and 16S with forward 16Sar-L (5′-CGCCTGTTTATCAAAAACAT-3′) and reverse 16Sbr-H (5′-CCG GTCTGAACTCAGATCACGT-3′) [43]. The amplification protocol consisted in an initial denaturation at 95 ℃ for 15 min, followed by 40 cycles (35 cycles for 16S) at 95 ℃ for 30 s, at 40 ℃ for 30 s (at 55 ℃ for 50 s for 16S), 72 for one minute 30 s (1 min for 16S) and a final step at 72 ℃ for 7 min. PCR product migration for 25 min at 180 V in a 1.5% agarose gel with SYBR Safe dye was read using a Gel Doc System (Bio-Rad, Hercules, USA). The amplicons were purified using Macherey Nagel plates (NucleoFast 96 PCR, Düren, Germany) and were sequenced using the same primers. The BigDye Terminator v1.1, v3.15 × Sequencing Buffer (Applied Biosystems, Warrington, UK) was run using an ABI 3100 automated sequencer (Applied Biosystems). The sequences obtained were assembled and analysed using the Chromas Pro v.1.77 software (Technelysium Pty. Ltd, Tewantin, Australia) and further queried against the National Center for Biotechnology Information (NCBI) online nucleotide database with the Basic Local Alignment Search Tool (BLAST) (http://blast.ncbi.nlm.nih.gov). A maximum likelihood phylogenetic tree was constructed with MEGA version 7.0.26 software [44, 45]. Statistical support of the internal tree branches was assessed by 1000 bootstrapping replicates.

### Data analysis

To determine differential peaks, spectral mass profiles with ClinProTools (Bruker Daltonics), representative peaks among the different groups were selected using several statistical tests, including the t-test, the analysis of variance test (ANOVA), Wilcoxon or Kruskal–Wallis (W/KW) tests, and the Anderson–Darling (AD) test. A *P*-value of 0.05 was set as the statistical significance cutoff [46]. A characteristic peak was considered when *P* < 0.05 in the AD test and *P*-value in the W/KW test was also < 0.05. When *P* = 0.05 in the AD test, a characteristic peak was selected if the corresponding *P*-value in the ANOVA was < 0.05 [47]. Informative peaks were those that were statistically significantly different between populations.

## Results

### Snail collection and morphological identification

A total of 182 snails collected from water bodies (rivers and temporary ponds) at different locations were selected for this study. These specimens, stored at -80 ℃, had previously been morphologically identified as belonging to the *Bi.* *pfeifferi* (*n* = 101) and *Bu.* *forskalii* (*n* = 81) species. The *Bi.* *pfeifferi* specimens originated from distinct geographical areas (Fig. 1b), notably in the NW in the SRD (*n* = 85) and in the SE in Kedougou, including both laboratory (LS) (*n* = 10) and wild strains (WS) (*n* = 6). *Bu.* *forskalii* was also found in the SRD (*n* = 77) and also in CS in the department of Diourbel (*n* = 4).

### MS identification of the two snail species

**Fig. 2:**
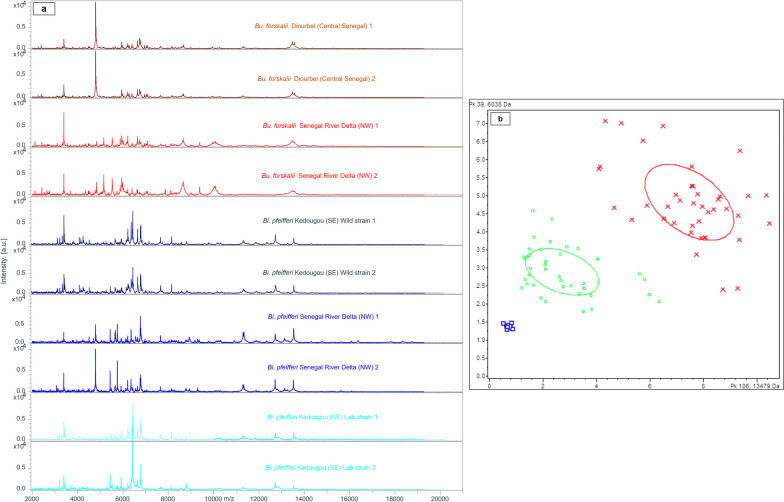
**a** Representative MS profiles of *Bu.* *forskalii* from Diourbel (1–2) and SRD (3–4) and *Bi.* *pfeifferi* from South-Eastern Senegal Wild strain (5, 6), lab strain (9–10) and *Bi.* *pfeifferi* from SRD (7–8) as a function of geographical location using FlexAnalysis v.3.3 software. au: arbitrary units; m/z: mass-to-charge ratio. **b** MALDI–TOF MS distinction of snail spectra using the feet shown on the PCA with ClinProTools software: *Bu.* *forskalii* (red crosses; ten specimens); *Bi.* *pfeifferi* (green circles; ten specimens) and out-group (*Planorbella* spp.) (blue squares; one specimen)

Intact *Bi.* *pfeifferi* (*n* = 101) and *Bu.* *forskalii* (*n* = 81) snail specimens were analysed by MALDI–TOF MS. Each specimen produced high-quality and reproducible spectra (Fig. 2a). The spectra obtained from each snail foot were then queried against the laboratory’s MALDI–TOF MS database which contained only reference spectra from snails collected in two areas of Senegal, namely Richard-Toll in the North and Niakhar in the Centre. In the first blind test, 92.1% (93/101) of *Bi.* *pfeifferi* specimens were successfully identified, with LSVs ranging from 1.78 to 2.48 [mean score ± standard deviation (*SD*) = 2.09 ± 0.20]. Eight of the 101 specimens yielded LSVs < 1.7 despite good-quality spectra. All eight specimens were re-included on the final blind test and successfully identified with LSVs ranging from 1.74 to 2.23.

Regarding *Bu.* *forskalii*, 98.8% (80/81) of specimens yielded LSVs ranging from 1.82 to 2.41 (mean score ± *SD*: 2.18 ± 0.13) on the first blind test. One of the 81 specimens, which originated from Diourbel, was identified as *Bu.* *senegalensis* (LSV = 1.83) and was excluded from the final blind test. Log score median and inter-quartile ranges were calculated for *Bi.* *pfeifferi* (median: 2.11, IQR: 1.99–2.19) and *Bu.* *forskalii* (median: 2.19, IQR: 2.10–2.28) (Table 1). Both the visual comparison of the spectra and the PCA performed with ClinProTools showed that the specimens of the same snail species clustered together (Fig. 2b).

**Table 1:** Detail of the results of the first blind test showing snail species identified with the pre-existing in-lab MALDI–TOF MS database and some specimens selected to update our reference database after DNA-based identification

Morphological ID	Geographical origin (colonies)	Number of specimens tested	Good-quality MS spectra	Number of specimens used for Blind test	Snail species ID by MS	LSV^a^ range [0–3]	Mean LSV (*SD*)^b^	Number identified with LSVs ≥ 1.7 (%)	Molecular identification (% of identity) (*n* = sequences)
*Bulinus* *forskalii*	Central Senegal (Diourbel)	4	4/4	4	*Bu.* *forskalii*	[2.24–2.39]	2.31 (0.05)	3 (75%)	*Bu.* *forskalii* (100%) (2)
	North-Western (Senegal River Delta)	77	77/77	77	*Bu.* *forskalii*	[1.82–2.41]	2.18 (0.01)	77 (100%)	*Bu.* *forskalii* (99.35%) (8)
*Biomphalaria* *pfeifferi*	North-Western (Senegal River Delta)	85	85/85	85	*Bi.* *pfeifferi*	[1.58–2.30]	2.09 (0.02)	77 (90.6%)	*Bi.* *pfeifferi* (100%) (8)
	South-Eastern (Laboratory strain)	10	10/10	10	*Bi.* *pfeifferi*	[2.17–2.48]	2.37 (0.03)	10 (100%)	*Bi.* *pfeifferi* (99.84%) (4)
	South-Eastern (Wild strain)	6	6/6	6	*Bi.* *pfeifferi*	[1.95–2.44]	2.21 (0.08)	6 (100%)	*Bi.* *pfeifferi* (99.82%) (3)
Total		182	182/182	182					25

^a^LSV: Log score value, ^b^*SD*: Standard deviation

### Intraspecific diversity in MS spectra of two snail species

**Fig. 3:**
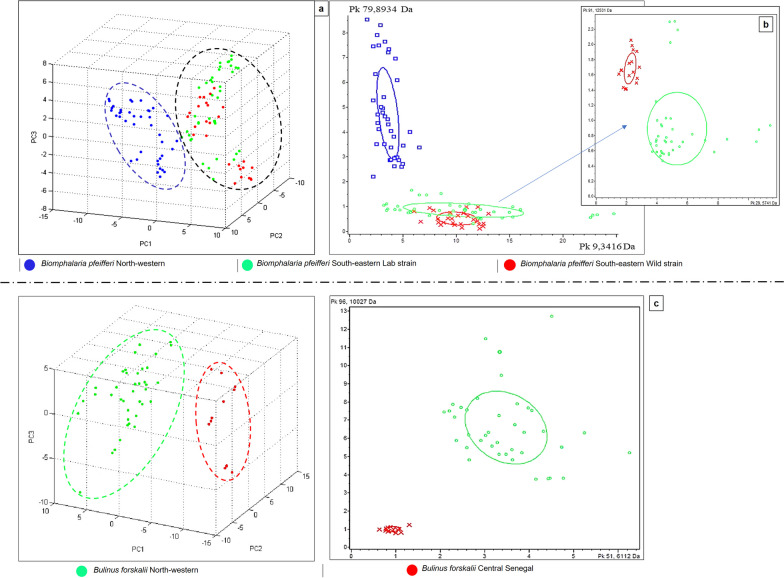
**a** PCA showing the clustering of MALDI-TOF MS spectra of *Bi.* *pfeifferi* specimens according to their geographical origin: *Bi.* *pfeifferi* from the Senegal River Delta in North-Western Senegal (blue), *Bi.* *pfeifferi* from Kedougou (South-Eastern Senegal) (green for laboratory strain and red for wild strain, (**b**) and (**c**) differentiation of two distinct *Bu.* *forskalii* populations: North-Western Senegal (green) and Central Senegal (red)

**Fig. 4:**
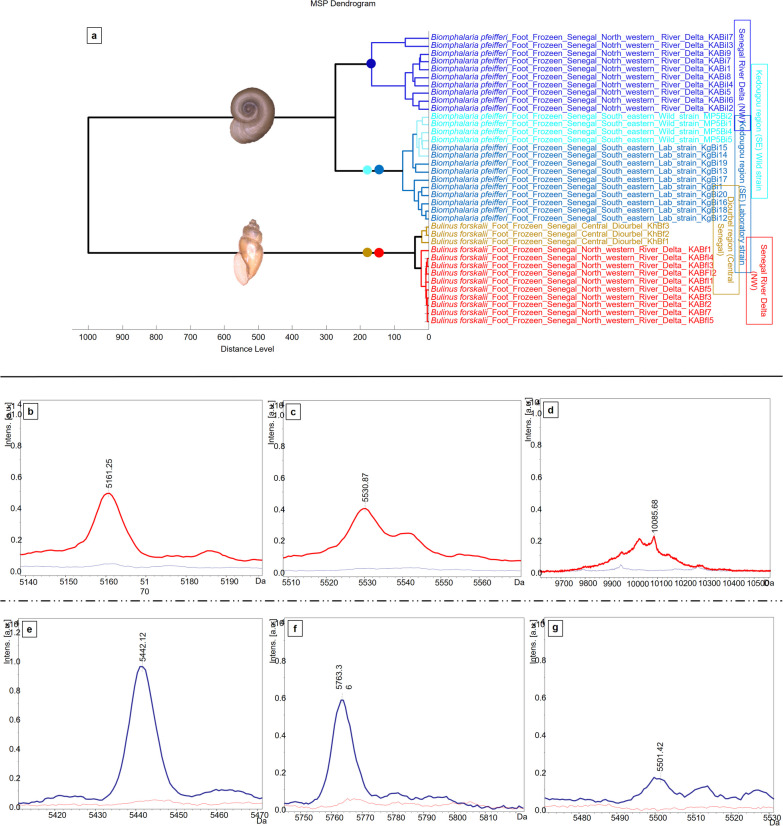
**a** Dendrogram based on a composite correlation index (CCI) matrix of spectra obtained from specimens of *Bi.* *pfeifferi* and *Bu.* *forskalii* selected at random from the four populations: ten spectra for *Bi.* *pfeifferi* and *Bu.* *forskalii* from the NW (SRD), ten for the laboratory strain *Bi.* *pfeifferi* from the SE (Kedougou), four for the wild strain and three spectra for *Bu.* *forskalii* from Central Senegal (Diourbel). **b–g** Characteristic peaks in the individual clean spectra of specimens from the north-west *Bu.* *forskalii* (red curves) and the north-west *Bi.* *pfeifferi* (blue curves) populations relative to specimens from central and South-Eastern Senegal, respectively, obtained by manual analysis in FlexAnalysis software: NW *Bu.* *forskalii* (**b**: 5161 Da, **c**: 5531 Da and **d**: 10,086 Da) and NW *Bi.* *pfeifferi* (**e**: 5442 Da, **f**: 5763 Da and **g**: 5501 Da). The x-axis indicates ionic mass values in Dalton (Da) and the y-axis indicates peak intensities expressed in arbitrary intensity units (a.u)

Visual observation of the spectral profiles obtained with the FlexAnalysis software did not allow for a clear differentiation between the *Bi.* *pfeifferi* populations originating from the north or the south of Senegal. Both the PCA (Fig. 3a) and the dendrogram (Fig. 4a) clearly separated these populations according to their geographical origin. A second PCA conducted only on the specimens collected in the SE area, highlighted a clear distinction between *Bi.* *pfeifferi* WS and LS (Fig. 3b).

PCA was applied to randomly selected *Bu.* *forskalii* snails collected either in the NW (SRD, *n* = 10) or CS (Diourbel, *n* = 3). Both the PCA (Fig. 3c) results and the dendrogram (Fig. 4a) clearly show the clustering of the spectra according to the geographical source of the *Bu.* *forskalii* specimens.

### Detection of peak biomarkers

Characteristic peaks were selected using the corresponding *P-*values obtained in the W/KW assay with the AD test displaying *P* < 0.05. The top ten best peaks are detailed in Table 2.

**Table 2:** Top ten discriminating MALDI–TOF MS peaks, selected with the ClinProTools software, for each group of snails

Index	Mass	DAve	Group A						Group B		
			PTTA	PWKW	PAD	Index	Mass	DAve	PTTA	PWKW	PAD
29	4802.21	76.48	< 10^–5^	< 10^–5^	< 10^–5^	36	5763.36^a^	31.43	< 10^–6^	< 10^–6^	< 10^–6^
30	4818	33.9	< 10^–4^	< 10^–5^	< 10^–3^	50	6439.1	30.89	< 10–6	< 10^–6^	< 10^–5^
35	5161.25 #	11.23	< 10^–4^	< 10^–5^	0.005	27	5442.12^a^	30.42	< 10^–6^	< 10^–6^	< 10^–5^
44	5881.69	9.82	< 10^–5^	< 10^–5^	0.003	6	3399.35	13.31	< 10^–6^	< 10^–5^	< 10^–4^
31	4832.72	9.81	0.002	0.007	0.006	21	4800.99	9.24	0.024	< 10^–6^	< 10^–6^
28	4776.29	9.67	< 10^–3^	< 10^–5^	< 10^–3^	7	3416.56	8.55	< 10^–6^	< 10^–6^	< 10^–4^
65	6650.44	7.99	< 10^–6^	< 10^–5^	0.012	1	3083.03	6.57	< 10^–4^	< 10^–6^	< 10^–6^
38	5530.87 #	7.19	0.002	< 10^–3^	< 10^–3^	77	8946.56	5.14	< 10^–6^	< 10^–6^	< 10^–6^
97	10,085.68 #	6.26	< 10^–5^	< 10^–5^	< 10^–3^	30	5501.42^a^	4.87	< 10^–6^	< 10^–6^	< 10^–6^
57	6276.78	6.19	< 10^–6^	< 10^–5^	< 10^–3^	76	8933.43	4.73	< 10^–6^	< 10^–6^	< 10^–6^

*Mass* m/z value, *DAve* Difference between the maxima land the minimal average peak intensity of all snail colonies, *PTTA* *t*-/analysis of variance test *P*-value, *PWKW* Wilcoxon/Kruskal–Wallis test *P*-value, *PAD* Anderson–Darling test *P*-value

^a^Characteristic peaks found only in colonies from the Senegal River Delta (NW), *Biomphalaria* *pfeifferi* NW and *Bulinus* *forskalii* NW

The ten selected peaks (ranging from 3083 to 10,086 m/z) displayed *P* < 0.05 with the W/KW test, indicating that they were informative and discriminative peaks in each group of snails. The GA tool of the ClinProTools software identified discriminating peaks between *Bu.* *forskalii* from the north and *Bu.* *forskalii* from CS, giving 100.00% CR and CV values. The same analysis was also performed to identify discriminating peaks between the mean profiles of *Bi.* *pfeifferi* NW and *Bi.* *pfeifferi* SE from Senegal. At least ten peaks were also selected from this group of *Bi.* *pfeifferi* with both 100.00% CR and CV. Furthermore, visual inspection of the spectra using FlexAnalysis software revealed at least three peaks in the *Bu.* *forskalii* NW population (5161 Da, 5531 Da, and 10,086 Da) and in *Bi.* *pfeifferi* NW (5442 Da, 5501 Da, and 5763 Da), which were not detected in Bu. forskalii CS and in *Bi.* *pfeifferi* SE from Senegal, respectively (Fig. 4b–g). The same analysis was also performed to identify discriminating peaks between the mean profiles of *Bi.* *pfeifferi* NW and *Bi.* *pfeifferi* SE from Senegal. At least ten peaks were also selected from this group of *Bi.* *pfeifferi* with both 100.00% CR and CV.

Furthermore, visual inspection of the spectra using FlexAnalysis software revealed at least three peaks in the *Bu.* *forskalii* NW population (5161 Da, 5531 Da, and 10,086 Da) and in *Bi.* *pfeifferi* NW (5442 Da, 5501 Da, and 5763 Da), which were not detected in *Bu.* *forskalii* CS and in *Bi.* *pfeifferi* SE from Senegal, respectively (Fig. 4b–g). These peaks were statistically significant and also detected with reproducible intensity in the mean spectrum of each group in ClinProTools (Table 2). All characteristic peaks obtained with ClinProTools software in both groups are available in Additional file 1: Tables S1, S2.

### DNA-based identification of snails

**Fig. 5:**
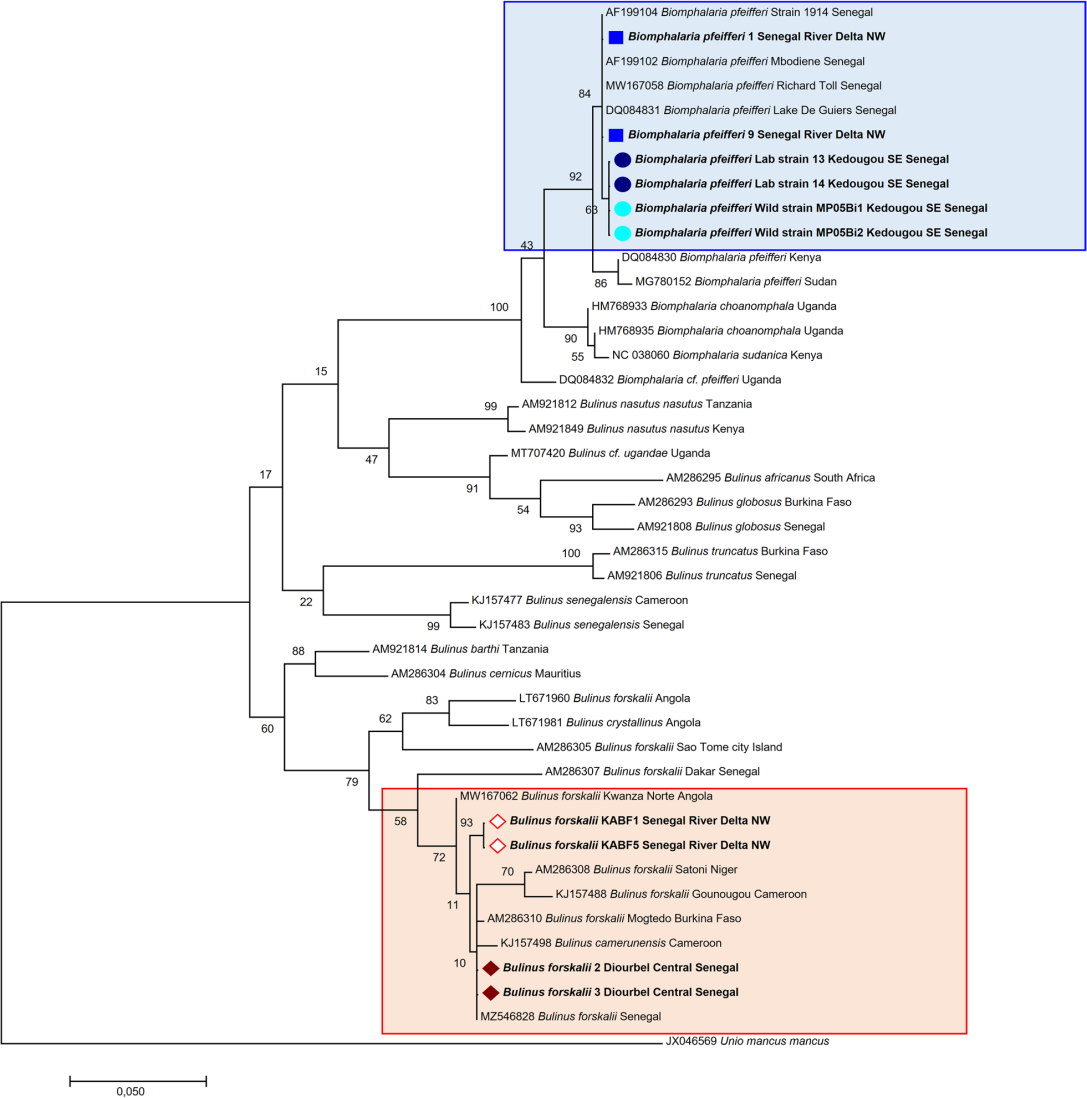
The tree was based on a nucleotide sequence of a 710 bp region of the cytochrome *c* oxidase subunit I (COI) gene (**a**) and the 550-bp 16S region (**b**), constructed using Kimura’s two-parameter distance-based maximum likelihood method with MEGA 7. The values on the branches are bootstrap support values based on 1000 replications. The branches have a pictogram corresponding to the bootstrap values. The identity of each taxon is encoded in bold with the pictogram according to the species

Nucleotide sequence analysis unambiguously confirmed the identity of these snail species using the COI region of the mitochondrial gene. BLAST analysis of COI sequences showed 99.82% to 100.00% identity with *Bi.* *pfeifferi* (*n* = 15) with GenBank accession numbers AF199099 and DQ084831, and 99.36% to 100.00% identity with *Bu.* *forskalii* (*n* = 10) with GB accession number MZ546828. The specimen from Diourbel (CS), identified as *Bu.* *senegalensis* (LSV = 1.83) by MALDI–TOF, was confirmed with 100% similarity to the sequence of *Bu.* *senegalensis* (Accession number OP811029) in GenBank. For 16S rRNA, three specimens from each population were used to confirm COI sequence-based identification, however, *Bu.* *forskalii* showed 96.42% to 96.68% identity with GenBank accession number AY029545, a specimen originating from Madagascar (there was no 16S sequence from *Bu.* *forskalii* originating in Senegal). Two phylogenetic trees, based on COI and 16S sequences, highlighted that *Bi.* *pfeifferi* from South-Eastern Senegal (in Kedougou) and North-Western Senegal (in the SRD) clustered in the same subgroup which includes sequences from GenBank of other *Bi.* *pfeifferi* specimens originating from Senegal. The same observation was made for the two *Bu.* *forskalii* populations (Fig. 5a, b). The *Bu.* *senegalensis* sequence found in the *Bu.* *forskalii* specimens from Diourbel was also introduced into the trees and groups with the homologous *Bu.* *senegalensis* sequences from GenBank. Representative sequences (COI and 16S rRNA) of *Bi.* *pfeifferi,* *Bu.* *forskalii* and *Bu.* *senegalensis* were deposited in the GenBank nucleotide database and are available in FASTA format in Additional file 2.

After molecular identification, new reference spectra of 15 snail specimens including ten *Bi.* *pfeifferi* and five *Bu.* *forskalii* from each of the geographical areas (NW, SE and CS) of Senegal were added to our in-laboratory MALDI–TOF MS reference spectra database. The final blind test against this updated database was carried out on the 166 remaining specimens (*Bi.* *pfeifferi*, *n* = 91, and *Bu.* *forskalii*, *n* = 75) enabling identification with LSVs ≥ 1.7 with a best-match reference of the same geographical source in 88% of the specimens. The average log score was significantly higher in the second blind test compared to the first blind test, with 2.24 ± 0.17 (*P* < 10^–8^) and 2.32 ± 0.19 (*P* < 10^–6^) for *Bi.* *pfeifferi* and *Bu.* *forskalii*, respectively. The respective medians show the same trends in both snail populations (median: 2.25, IQR: 2.15–2.33 vs. median: 2.33, IQR: 2.19–2.43) (Additional file 1: Fig. S1). Overall, 166 specimens were submitted to the second blind test with 100% (166/166) identified to species level. As detailed in Table 3, the geographical source of these specimens was correctly inferred in 146 (88%) of the specimens overall, including 90.1% (82/91) in *Bi.* *pfeifferi* and 85.3% (64/75) in *Bu.* *forskalii*.

**Table 3:** Detail of the final blind test results, after updating the reference spectra database with the spectra of 15 specimens

Morphological ID	Geographical origin	Number of specimens tested	Spectra added to database	Number of specimens used for Second blind test	Snail species ID by MS	LSV range [0–3]	Mean LSV (*SD*)^b^	Snail species ID by origin
*Bulinus* *forskalii*	Central Senegal (Diourbel)	3	1	2	*Bu.* *forskalii*	[2.40–2.58]	2.51 (0.06)	100.0% (2/2)
	North-Western (Senegal River Delta)	77	4	73	*Bu.* *forskalii*	[1.82–2.70]	2.32 (0.02)	84.9% (62/73)
*Biomphalaria* *pfeifferi*	North-Western (Senegal River Delta)	85	4	81	*Bi.* *pfeifferi*	[1.74–2.63]	2.21 (0.02)	90.1% (73/81)
	South-Eastern (Kedougou) (Laboratory strain)	10	3	7	*Bi.* *pfeifferi*	[2.17–2.61]	2.42 (0.04)	100.0% (7/7)
	South-Eastern (Kedougou) (Wild strain)	6	3	3	*Bi.* *pfeifferi*	[1.90–2.61]	2.30 (0.10)	66.7% (2/3)
Total		181	15	166				

^a^LSV: Log score value; ^b^*SD*: Standard deviation

## Discussion

Recent studies have shown that MALDI–TOF spectrometry is a powerful, rapid and inexpensive tool for the identification of arthropods, snails, including marine bivalves [30], and some species of the gastropod family [31, 48]. The main result of this study is the evidence that MALDI–TOF MS has not only the capability to identify and discriminate between distinct species, but also to trace the geographical source of intraspecific populations of freshwater gastropod snails involved in *Schistosoma* spp. transmission.

In line with many other studies [49, 50], the critical factor for success was the addition of new spectra of snail specimens to our database. In our study, we had eight specimens of *Bi.* *pfeifferi* from the South-East that were not identified in the first blind test and were correctly identified in the final blind test. Indeed, increasing the number of reference spectra to the database allowed for higher LSVs and enhanced the accuracy, which in turn allowed the geographical source to be determined. Similar observations have been reported in a study conducted on bed bugs of different geographical source [51]. Cluster analysis made it possible to distinguish between bed bugs specimens with different geographical sources, in particular *Cimex* *lectularius* from Germany and London [51]. Furthermore, Raharimalala et al. [52] reported variations in the MS profile of mosquitoes depending on the area where they had been collected, and Fall et al*.* [53] demonstrated the capability of MALDI–TOF to differentiate between different geographical sources within intraspecific female mosquitoes. The different MALDI–TOF MS spectra acquired from *Bi.* *pfeifferi* and *Bu.* *forskalii* individuals according to their geographical source may result from evolutionary processes involved in species separation due to adaptation to heterogeneous environmental conditions, which may influence the proteome of these freshwater snails by modifying their biotopes or even their microbiome. Furthermore, the observation of several differential peaks between specimens within populations of *Bu.* *forskalii* and *Bi.* *pfeifferi* reinforces the hypothesis that the protein composition between specimens of the same species differs depending on their geographic source, as suggested by the study by Fall et al. [53] performed on *Aedes* *aegypti* and in *Ae.* *polynesiensis* mosquito populations. The ClinPro Tools software, via the Genetic Algorithm function, allowed us to obtain different peaks discriminating between specimens according to their locality. However, this approach does not enable the precise identification of the biomolecules involved in the MS spectra or in the acquisition of these peaks and may require other proteomic tools or approaches.

Our nucleotide-based phylogenetic analysis revealed a clear separation between the *Bi.* *pfeifferi* and *Bu.* *forskalii* snail species, however, it did not discriminate between the populations of distinct geographical sources. The field and laboratory *Bi.* *pfeifferi* specimens (SE of Senegal) were neither clearly distinguished by the nucleotide-based phylogenetic tree nor by the global PCA. This indicates a relatively homogeneous genetic background of the SE specimens that is distinct from the NW specimens. In contrast, the PCA carried out on the MALDI–TOF MS spectra of the *Bi.* *pfeifferi* specimens originating only from South-Eastern Senegal, clearly distinguished wild from laboratory strains. This finding suggests that beyond genetic factors, environmental factors influence the MALDI–TOF MS spectra of *Bi.* *pfeifferi*. Indeed, laboratory rearing conditions contrast sharply from those in the field. This dependence on environmental factors could be interpreted as one limitation of using MALDI–TOF MS as a substitute for genetic analysis tools. In contrast, however, in 2019 Karger et al*.* [54] suggested that MALDI–TOF MS enables phenotypic analysis targeting proteins likely to reveal interactions with the environment that are often undetectable in genetic analyses.

In this study, MALDI–TOF could also differentiate *Bu.* *senegalensis* from *Bu.* *forskalii*. Previous studies have highlighted the misidentification between these two species, due to their similar morphological characteristics and their frequent sympatry [55].This reinforces the idea that this rapid, inexpensive, high-throughput approach does not require any malacological expertise in snail identification, especially as MALDI–TOF was used here to discriminate between *Bu.* *forskalii* and its closely related species *Bu.* *senegalensis*.

The main limitation of our study is the relatively small number of snail samples and collection points, and the collection point heterogeneity. Indeed, collection point were very small in some areas, notably in the centre of the country at Niakhar, compared with the Senegal River Delta (North). Including a higher number of specimens in each area and a higher number of collection sites, including sites located in the Eastern and Western parts of the country, would have enabled us to better assess the protein fingerprinting heterogeneity according to locality.

## Conclusions

Our results demonstrated for the first time that MALDI–TOF MS is a suitable tool for identifying and differentiating snail populations according to their geographical origin. This tool shows a greater resolution compared to morphological and molecular tools, as it makes it possible to discriminate between populations with different geographical origins. In the near future, this tool is likely to revolutionise epidemiological studies in areas which are endemic for schistosomiasis. In the context of pre-eliminating schistosomiasis, it is critical to understand the dynamics of the intermediate snail host populations in endemic areas. The use of MALDI–TOF MS opens the way for future studies to improve schistosomiasis control strategies. Further research will aim to assess whether MALDI–TOF MS can identify parasitised snails and further dissect the mechanisms of host-parasite compatibility.

### Supplementary Information

**Additional file 1: Abbreviations list. Table S1**. Characteristic peaks, obtained by ClinProTools software for group A (population 1 = *Bu.* *forskalii* NW samples and population 2 = *Bu.* *forskalii* Central Senegal samples). **Table S2**. Characteristic peaks, obtained by ClinProTools software for group B (population 3 = *Bi.* *pfeifferi* NW samples and population 4 = *Bi.* *pfeifferi* SE lab strain and wild strain samples). **Fig. S1**. Graphic representation showing the classification of the LSVs of the first and second blind test according to the species of *Biomphalaria* *pfeifferi* and *Bulinus* *forskalii.*

**Additional file 2:** Representative sequences of *Bi.* *pfeifferi* and *Bu.* *forskalii* deposited in GenBank.

## Data Availability

All data from this study are reported in the manuscript and in the supplementary data. COI sequences of *Bulinus* *forskalii* snails from the SRD and Diourbel regions have been deposited in GenBank under accession numbers OM535893, OM535894, OM535895, ON077052, and ON077053, respectively, as well as those of *Biomphalaria* *pfeifferi* from Kedougou (GenBank accession numbers: OM535896 and OM535897). The 16S rRNA sequences of *Bulinus* *forskalii* from the SRD and Diourbel have been deposited in GenBank under the following accession numbers: ON062292, ON062293, ON062294, ON062295, ON062296, and ON062297, respectively. The sequences in FASTA format of all sequenced specimens are available in the supplementary data.

## Abbreviations

MALDI–TOF MS: Matrix-assisted laser desorption/ionization time-of-flight mass spectrometry
NTD: Neglected tropical disease
WHO: World Health Organization
SRD: Senegal River Delta
CS: Central Senegal
NW: North-West
SE: Souht-East
LSV: Log score value
*SD*: Standard deviation
