# Supplementary material for: Matrix-assisted laser desorption/ionization time-of-flight mass spectrometry traces the geographical source of Biomphalaria pfeifferi and Bulinus forskalii, involved in schistosomiasis transmission
Source: Infect Dis Poverty. 2024 Jan 29;13:11. doi: 10.1186/s40249-023-01168-y (PMC10823745; doi:10.1186/s40249-023-01168-y)
Supplement: Supplementary file 1 — Additional file 1: Abbreviations list. Table S1. Characteristic peaks, obtained by ClinProTools software for group A (population 1 = Bu. forskalii NW samples and population 2 = Bu. forskalii Central Senegal samples). Table S2. Characteristic peaks, obtained by ClinProTools software for group B (population 3 = Bi. pfeifferi NW samples and population 4 = Bi. pfeifferi SE lab strain and wild strain samples). Fig. S1. Graphic representation showing the classification of the LSVs of the first and second blind test according to the species of Biomphalaria pfeifferi and Bulinus forskalii. [file 40249_2023_1168_MOESM1_ESM.docx]

**Additional file 1**

**Abbreviations list**

ACN Acetonitrile

AD Anderson-Darling

ANOVA Analysis of variance

BLAST Basic Local Alignment Search Tool

CCI Composite correlation index

CHCA α-Cyano-4- hydroxycinnamic acid

COI Cytochrome oxidase subunit I

CS Central Senegal

CV Cross validation

DNA Deoxyribonucleic acid

EDTA Ethylenediaminetetraacetic acid

HPLC High-performance liquid chromatography

GA Genetic Algorithm

IQR Inter-quartile ranges

kDa KiloDalton

LS Laboratory Strain

LSV Log Score value

MALDI-TOF MS Matrix-assisted laser desorption/ionization time-of-flight mass spectrometry

MEGA Molecular Evolutionary Genetics Analysis

NCBI National Centre for Biotechnology Information

NTD Neglected Tropical Disease

NW North-West

PCA Principal Component Analysis

PCR Polymerase Chain Reaction

PPM Parts Per Million

QGIS Quantum Geological Information System

RC Recognition capability

RNA Ribonucleic acid

SD Standard Deviation

SE South-East

SRD Senegal River Delta

WHO World Health Organization

WS Wild Strain

W/KW Wilcoxon or Kruskal-Wallis

**Table S1.** Characteristic peaks, obtained by ClinProTools software for group A (population 1= *Bu. forskalii* NW samples and population 2= *Bu. forskalii* Central Senegal samples).

**Table S2.** Characteristic peaks, obtained by ClinProTools software for group B (population 3= *Bi. pfeifferi* NW samples and population 4= *Bi. pfeifferi* SE lab strain and Wild strain samples).

**Fig. S1.** Graphical representation showing the classification of the LSVs of the first and second blind test according to the species of *Biomphalaria pfeifferi* and *Bulinus forskalii*

**Table S1.** Characteristic peaks, obtained by ClinProTools software for group A (population 1= *Bu. forskalii* NW samples and population 2= *Bu. forskalii* Central Senegal samples).

| **Index** | **Mass** | **DAve** | **PTTA** | **PWKW** | **PAD** |
| --- | --- | --- | --- | --- | --- |
| 1 | 2108.38 | 2.24 | 0.0141 | 0.00000296 | < 0.000001 |
| 2 | 2262.89 | 3.56 | 0.000425 | 0.00000296 | 0.00083 |
| 3 | 2398.83 | 5.84 | 0.000134 | 0.00000296 | 0.000262 |
| 4 | 2406.59 | 3.03 | 0.0000153 | 0.00000296 | 0.0241 |
| 5 | 2429.71 | 3.09 | 0.000324 | 0.000402 | 0.00704 |
| 6 | 2445.02 | 1.21 | 0.0197 | 0.0289 | 0.00222 |
| 7 | 3108.76 | 1.17 | 0.000329 | 0.000402 | 0.966 |
| 8 | 3324.48 | 2.4 | 0.00000653 | 0.00000296 | 0.0684 |
| 9 | 3357.84 | 0.61 | 0.063 | 0.0787 | 0.6 |
| 10 | 3376.89 | 0.03 | 0.934 | 0.649 | 0.387 |
| 11 | 3384.74 | 0.03 | 0.945 | 0.879 | 0.319 |
| 12 | 3399.88 | 1.77 | 0.478 | 0.649 | 0.121 |
| 13 | 3415.59 | 4.51 | < 0.000001 | 0.00000296 | 0.0556 |
| 14 | 3430.44 | 1.1 | 0.000144 | 0.0000159 | 0.514 |
| 15 | 3446.14 | 0.63 | 0.00298 | 0.0000888 | 0.0317 |
| 16 | 3479.29 | 1.27 | < 0.000001 | 0.00000296 | 0.158 |
| 17 | 3679.32 | 2.24 | 0.0000173 | 0.000166 | 0.0556 |
| 18 | 3775.74 | 2.65 | 0.000582 | 0.00000296 | 0.000202 |
| 19 | 4085.3 | 1.54 | 0.0000173 | 0.0000605 | 0.354 |
| 20 | 4320.05 | 0.06 | 0.799 | 0.879 | 0.238 |
| 21 | 4332.49 | 0.66 | 0.0271 | 0.0337 | 0.881 |
| 22 | 4347.17 | 1.94 | 0.0003 | 0.000119 | 0.055 |
| 23 | 4476.46 | 2.11 | 0.000123 | 0.000402 | 0.0058 |
| 24 | 4495.56 | 0.93 | 0.00603 | 0.00301 | 0.651 |
| 25 | 4502.8 | 0.84 | 0.0225 | 0.00154 | 0.591 |
| 26 | 4524.92 | 0.87 | 0.0136 | 0.0522 | 0.768 |
| 27 | 4549.41 | 0.04 | 0.926 | 0.948 | 0.257 |
| 28 | 4776.29 | 9.67 | 0.000144 | 0.00000296 | 0.000679 |
| 29 | 4802.21 | 76.48 | 0.0000783 | 0.00000296 | 0.0000338 |
| 30 | 4818 | 33.9 | 0.0000931 | 0.00000296 | 0.000285 |
| 31 | 4832.72 | 9.81 | 0.00167 | 0.00722 | 0.00636 |
| 32 | 4975.57 | 0.24 | 0.224 | 0.319 | 0.355 |
| 33 | 5085.76 | 1.47 | 0.000116 | 0.000233 | 0.0422 |
| 34 | 5143.92 | 2.14 | 0.000134 | 0.0000392 | 0.0422 |
| 35 | 5161.25 | 11.23 | 0.0000511 | 0.00000296 | 0.00532 |
| 36 | 5176.4 | 1.74 | 0.0016 | 0.00154 | 0.109 |
| 37 | 5285.9 | 0.49 | 0.0199 | 0.0215 | 0.108 |
| 38 | 5530.87 | 7.19 | 0.0016 | 0.000166 | 0.000149 |
| 39 | 5540.38 | 4.02 | 0.00124 | 0.00242 | 0.0422 |
| 40 | 5595.99 | 0.07 | 0.826 | 0.77 | 0.091 |
| 41 | 5655.28 | 1.91 | 0.00091 | 0.00154 | 0.252 |
| 42 | 5675.46 | 0.56 | 0.242 | 0.379 | 0.22 |
| 43 | 5741.05 | 2.04 | 0.00000286 | 0.0000159 | 0.0269 |
| 44 | 5881.69 | 9.82 | 0.0000268 | 0.00000296 | 0.00305 |
| 45 | 5897.64 | 2.86 | 0.00000499 | 0.00000296 | 0.26 |
| 46 | 5953.77 | 1.61 | 0.554 | 0.986 | 0.082 |
| 47 | 5981.59 | 1.42 | 0.255 | 0.216 | 0.464 |
| 48 | 6010.32 | 1.57 | 0.0172 | 0.0522 | 0.0305 |
| 49 | 6035.18 | 2.88 | 0.0000511 | 0.000119 | 0.0129 |
| 50 | 6109.18 | 2.64 | < 0.000001 | 0.00000296 | 0.00083 |
| 51 | 6138.64 | 0.94 | 0.00122 | 0.000402 | 0.102 |
| 52 | 6162.91 | 0.08 | 0.866 | 1 | 0.177 |
| 53 | 6183.46 | 1.43 | 0.000781 | 0.000707 | 0.0269 |
| 54 | 6206.76 | 0.64 | 0.031 | 0.101 | 0.116 |
| 55 | 6220.69 | 3.83 | 0.00000594 | 0.0000159 | 0.0947 |
| 56 | 6235.81 | 3.67 | < 0.000001 | 0.00000296 | 0.00222 |
| 57 | 6276.78 | 6.19 | < 0.000001 | 0.00000296 | 0.000941 |
| 58 | 6372.55 | 0.07 | 0.81 | 0.879 | 0.355 |
| 59 | 6401.45 | 0.41 | 0.6 | 0.582 | 0.126 |
| 60 | 6420.05 | 0.74 | 0.02 | 0.0215 | 0.493 |
| 61 | 6443.53 | 1.17 | 0.00331 | 0.0105 | 0.0991 |
| 62 | 6463.4 | 0.55 | 0.268 | 0.948 | 0.00107 |
| 63 | 6553.43 | 1.27 | 0.00000682 | 0.000119 | 0.0369 |
| 64 | 6629.1 | 0.35 | 0.534 | 0.918 | 0.0327 |
| 65 | 6650.44 | 7.99 | < 0.000001 | 0.00000296 | 0.0118 |
| 66 | 6741.99 | 11.19 | < 0.000001 | 0.00000296 | 0.0684 |
| 67 | 6787.08 | 8.95 | 0.0000268 | 0.0000254 | 0.257 |
| 68 | 6879.82 | 1.08 | 0.0016 | 0.00301 | 0.125 |
| 69 | 6960.4 | 3.94 | 0.00000653 | 0.00000296 | 0.347 |
| 70 | 7047.6 | 3.11 | < 0.000001 | 0.00000296 | 0.149 |
| 71 | 7088.87 | 1.7 | 0.0175 | 0.0459 | 0.149 |
| 72 | 7194.08 | 1.15 | 0.0000013 | 0.00000296 | 0.0792 |
| 73 | 7661.43 | 1.25 | 0.0174 | 0.0215 | 0.387 |
| 74 | 7755.95 | 1.11 | 0.00000965 | 0.00000296 | 0.0131 |
| 75 | 7870.29 | 3.04 | 0.000815 | 0.0000101 | 0.0000199 |
| 76 | 8114.95 | 2.31 | 0.00018 | 0.000402 | 0.0327 |
| 77 | 8138.11 | 0.04 | 0.86 | 0.809 | 0.355 |
| 78 | 8170.04 | 1.86 | 0.0000512 | 0.000119 | 0.218 |
| 79 | 8217.53 | 0.58 | 0.0194 | 0.0289 | 0.177 |
| 80 | 8264.95 | 0.97 | 0.0496 | 0.142 | 0.0423 |
| 81 | 8309.85 | 0.12 | 0.395 | 0.546 | 0.89 |
| 82 | 8348.53 | 1.11 | 0.0000742 | 0.00000573 | 0.0305 |
| 83 | 8436.8 | 0.4 | 0.151 | 0.127 | 0.354 |
| 84 | 8451.36 | 0.66 | 0.014 | 0.0105 | 0.218 |
| 85 | 8511.34 | 0.81 | 0.0123 | 0.0253 | 0.00636 |
| 86 | 8577.99 | 1.67 | 0.00223 | 0.000707 | 0.0229 |
| 87 | 8637.52 | 2.89 | 0.00326 | 0.00194 | 0.00642 |
| 88 | 8662.4 | 4.42 | 0.000835 | 0.00242 | 0.252 |
| 89 | 8688.28 | 4.14 | 0.000106 | 0.000402 | 0.0991 |
| 90 | 9003.7 | 0.87 | 0.000533 | 0.0000101 | 0.0878 |
| 91 | 9072.76 | 0.62 | 0.0014 | 0.000119 | 0.022 |
| 92 | 9263.13 | 1.2 | 0.00000343 | 0.00000296 | 0.241 |
| 93 | 9392.57 | 3.19 | 0.000691 | 0.0000254 | 0.0129 |
| 94 | 9794.16 | 0.61 | 0.0000297 | 0.0000159 | 0.904 |
| 95 | 9950.05 | 2.23 | < 0.000001 | 0.00000296 | 0.00283 |
| 96 | 10027.34 | 5.78 | < 0.000001 | 0.00000296 | 0.000312 |
| 97 | 10085.68 | 6.26 | 0.00000171 | 0.00000296 | 0.000475 |
| 98 | 10143.05 | 3.09 | < 0.000001 | 0.00000296 | 0.00067 |
| 99 | 10172.35 | 2.15 | < 0.000001 | 0.00000296 | 0.00331 |
| 100 | 10268.06 | 0.2 | 0.323 | 0.473 | 0.904 |
| 101 | 11178.64 | 0.4 | 0.0109 | 0.00722 | 0.0083 |
| 102 | 11306.08 | 0.26 | 0.686 | 0.473 | 0.987 |
| 103 | 11346.26 | 0.86 | 0.151 | 0.77 | 0.000325 |
| 104 | 11741.53 | 0.28 | 0.0175 | 0.0289 | 0.848 |
| 105 | 11778.14 | 0.2 | 0.138 | 0.158 | 0.864 |
| 106 | 11865.07 | 0.11 | 0.47 | 0.444 | 0.252 |
| 107 | 12064.64 | 0.56 | 0.0000778 | 0.0000101 | 0.0327 |
| 108 | 12323.67 | 0.37 | 0.00635 | 0.0105 | 0.0794 |
| 109 | 12984.97 | 0.37 | 0.0000395 | 0.0000254 | 0.881 |
| 110 | 13177.34 | 1.02 | 0.0000108 | 0.00000296 | 0.177 |
| 111 | 13277.26 | 0.55 | 0.00309 | 0.000929 | 0.0326 |
| 112 | 13479.03 | 3.16 | 0.00189 | 0.00194 | 0.0436 |
| 113 | 13569.38 | 2.21 | 0.0466 | 0.0787 | 0.28 |
| 114 | 13906.12 | 0.52 | 0.031 | 0.0892 | 0.00549 |
| 115 | 14383.15 | 0.49 | 0.00000897 | 0.0000101 | 0.493 |
| 116 | 14955.88 | 0.11 | 0.348 | 0.216 | 0.125 |
| 117 | 15301.16 | 0.5 | 0.0663 | 0.113 | 0.0000391 |
| 118 | 16061.35 | 0.42 | 0.00173 | 0.00054 | 0.0385 |
| 119 | 16421.82 | 1.07 | 0.0106 | 0.00032 | 0.00000495 |
| 120 | 16612.08 | 0.01 | 0.926 | 0.618 | 0.238 |

DAve: Difference between the maxima land the minimal average peak intensity of all snails populations.
PTTA: *P*-value obtained throught-test. PWKW : *P*-value obtained through Wilcoxon/Kruskal-Wallis test. PAD : *P*-value obtained through Anderson-Darling test.

**Table S2.** Characteristic peaks, obtained by ClinProTools software for group B (population 3= *Bi. pfeifferi* NW samples and population 4= *Bi. pfeifferi* SE lab strain and Wild strain samples).

| **Index** | **Mass** | **DAve** | **PTTA** | **PWKW** | **PAD** |
| --- | --- | --- | --- | --- | --- |
| 1 | 3083.03 | 6.57 | 0.0000399 | < 0.000001 | < 0.000001 |
| 2 | 3108.62 | 1.14 | 0.00224 | 0.0072 | 0.00494 |
| 3 | 3218.35 | 4.05 | < 0.000001 | < 0.000001 | 0.00378 |
| 4 | 3325.13 | 3.23 | < 0.000001 | < 0.000001 | 0.0000679 |
| 5 | 3385.99 | 2.84 | < 0.000001 | < 0.000001 | < 0.000001 |
| 6 | 3399.35 | 13.31 | < 0.000001 | 0.00000725 | 0.0000196 |
| 7 | 3416.56 | 8.55 | < 0.000001 | < 0.000001 | 0.0000357 |
| 8 | 3432.99 | 3.89 | < 0.000001 | < 0.000001 | 0.00494 |
| 9 | 3446 | 2.24 | < 0.000001 | < 0.000001 | 0.00205 |
| 10 | 3674.65 | 0.87 | 0.0426 | 0.00226 | < 0.000001 |
| 11 | 3765.32 | 1.69 | 0.0002 | < 0.000001 | < 0.000001 |
| 12 | 3773.77 | 0.18 | 0.346 | 0.914 | 0.000393 |
| 13 | 3816.94 | 2.43 | < 0.000001 | < 0.000001 | 0.0000176 |
| 14 | 3830.91 | 1.1 | < 0.000001 | < 0.000001 | 0.0652 |
| 15 | 4076.62 | 2.2 | < 0.000001 | < 0.000001 | < 0.000001 |
| 16 | 4145.57 | 1.27 | < 0.000001 | 0.0000135 | < 0.000001 |
| 17 | 4215.43 | 0.82 | 0.003 | 0.0022 | 0.0109 |
| 18 | 4243.83 | 1.99 | < 0.000001 | 0.00000883 | 0.0000522 |
| 19 | 4262.5 | 0.28 | 0.389 | 0.635 | 0.000182 |
| 20 | 4515.03 | 1.15 | 0.00655 | 0.0865 | 0.0000436 |
| 21 | 4800.99 | 9.24 | 0.0244 | < 0.000001 | < 0.000001 |
| 22 | 4818.61 | 3.3 | 0.000332 | < 0.000001 | < 0.000001 |
| 23 | 5174.76 | 1.73 | 0.0000729 | < 0.000001 | < 0.000001 |
| 24 | 5202.39 | 1.48 | 0.000309 | 0.0000492 | < 0.000001 |
| 25 | 5286.66 | 2.85 | 0.00000394 | 0.00000389 | < 0.000001 |
| 26 | 5423.78 | 1.58 | 0.0000153 | 0.0000884 | < 0.000001 |
| 27 | 5442.12 | 30.42 | < 0.000001 | < 0.000001 | < 0.000001 |
| 28 | 5463.47 | 2.97 | < 0.000001 | < 0.000001 | < 0.000001 |
| 29 | 5484.74 | 0.84 | 0.00454 | 0.000443 | 0.000264 |
| 30 | 5501.42 | 4.87 | < 0.000001 | < 0.000001 | < 0.000001 |
| 31 | 5514.27 | 2.51 | < 0.000001 | < 0.000001 | < 0.000001 |
| 32 | 5528.99 | 2.09 | 0.00000402 | < 0.000001 | < 0.000001 |
| 33 | 5655.29 | 1.3 | 0.0772 | 0.0531 | 0.000203 |
| 34 | 5674.21 | 0.44 | 0.564 | 0.249 | 0.00124 |
| 35 | 5738.48 | 0.55 | 0.037 | 0.0332 | 0.123 |
| 36 | 5763.36 | 31.43 | < 0.000001 | < 0.000001 | < 0.000001 |
| 37 | 5780.1 | 2.32 | 0.00000274 | < 0.000001 | 0.0000166 |
| 38 | 5794.34 | 0.12 | 0.641 | 0.914 | 0.0142 |
| 39 | 5839.5 | 3.35 | 0.000053 | 0.0509 | < 0.000001 |
| 40 | 5901.66 | 2.96 | < 0.000001 | < 0.000001 | 0.0000444 |
| 41 | 5924.54 | 4.19 | 0.00000471 | 0.00231 | 0.00000276 |
| 42 | 5944.57 | 2.08 | < 0.000001 | < 0.000001 | 0.00000128 |
| 43 | 5980.04 | 1.98 | < 0.000001 | < 0.000001 | < 0.000001 |
| 44 | 6041.94 | 0.37 | 0.166 | 0.854 | 0.000031 |
| 45 | 6176.96 | 0.68 | 0.0138 | 0.00874 | 0.91 |
| 46 | 6220.66 | 2.35 | 0.0245 | 0.067 | 0.261 |
| 47 | 6238.23 | 3.66 | < 0.000001 | < 0.000001 | 0.00000554 |
| 48 | 6252.1 | 2.03 | < 0.000001 | < 0.000001 | 0.0115 |
| 49 | 6365.85 | 8.18 | < 0.000001 | < 0.000001 | 0.0666 |
| 50 | 6439.1 | 30.89 | < 0.000001 | < 0.000001 | 0.00000123 |
| 51 | 6457.99 | 4.47 | < 0.000001 | < 0.000001 | 0.00494 |
| 52 | 6575.38 | 0.49 | 0.073 | 0.0068 | < 0.000001 |
| 53 | 6651.41 | 4.38 | < 0.000001 | 0.000617 | 0.00028 |
| 54 | 6670.18 | 0.18 | 0.617 | 0.914 | 0.00718 |
| 55 | 6772.69 | 2.26 | 0.194 | 0.247 | 0.000222 |
| 56 | 6794.4 | 1.71 | 0.194 | 0.396 | 0.00873 |
| 57 | 6842.35 | 0.14 | 0.56 | 0.914 | 0.0289 |
| 58 | 6980.43 | 0.33 | 0.116 | 0.0285 | 0.00124 |
| 59 | 7058.67 | 1.3 | < 0.000001 | < 0.000001 | 0.0212 |
| 60 | 7157.84 | 1.82 | 0.0000778 | 0.00000404 | 0.0000679 |
| 61 | 7605.44 | 0.86 | < 0.000001 | 0.0000371 | 0.00191 |
| 62 | 7660.1 | 1.36 | 0.0118 | 0.0121 | 0.0088 |
| 63 | 7736.55 | 0.35 | 0.0151 | 0.0991 | 0.00587 |
| 64 | 8045.09 | 0.91 | 0.0166 | 0.0000113 | < 0.000001 |
| 65 | 8151.79 | 1.84 | 0.000109 | 0.0563 | 0.00000217 |
| 66 | 8218.12 | 0.42 | 0.00119 | 0.00123 | 0.005 |
| 67 | 8291.62 | 1.69 | < 0.000001 | < 0.000001 | < 0.000001 |
| 68 | 8358.25 | 3.13 | 0.00000197 | < 0.000001 | < 0.000001 |
| 69 | 8450.85 | 1.01 | < 0.000001 | < 0.000001 | 0.0467 |
| 70 | 8566.56 | 0.2 | 0.16 | 0.0563 | 0.0766 |
| 71 | 8596.73 | 0.54 | 0.00136 | 0.00449 | 0.282 |
| 72 | 8699.88 | 1.17 | 0.00000645 | < 0.000001 | < 0.000001 |
| 73 | 8771.16 | 3.26 | < 0.000001 | < 0.000001 | < 0.000001 |
| 74 | 8804.45 | 0.8 | 0.0356 | 0.022 | 0.00571 |
| 75 | 8827.93 | 4.66 | < 0.000001 | < 0.000001 | < 0.000001 |
| 76 | 8933.43 | 4.73 | < 0.000001 | < 0.000001 | < 0.000001 |
| 77 | 8946.56 | 5.14 | < 0.000001 | < 0.000001 | < 0.000001 |
| 78 | 8971.05 | 3.75 | < 0.000001 | < 0.000001 | < 0.000001 |
| 79 | 8997.88 | 1.46 | < 0.000001 | < 0.000001 | < 0.000001 |
| 80 | 9300.84 | 1.14 | 0.000604 | < 0.000001 | < 0.000001 |
| 81 | 9326.23 | 2.82 | < 0.000001 | < 0.000001 | < 0.000001 |
| 82 | 9768.06 | 1.43 | 0.0000149 | < 0.000001 | < 0.000001 |
| 83 | 9840.51 | 0.74 | < 0.000001 | < 0.000001 | 0.000012 |
| 84 | 9898.67 | 0.44 | < 0.000001 | < 0.000001 | 0.604 |
| 85 | 10024.58 | 1.06 | < 0.000001 | < 0.000001 | < 0.000001 |
| 86 | 10092.45 | 2.99 | < 0.000001 | < 0.000001 | < 0.000001 |
| 87 | 10125.39 | 0.39 | 0.0000637 | 0.00014 | 0.524 |
| 88 | 10156.87 | 0.35 | 0.0467 | 0.0424 | 0.0298 |
| 89 | 10216.27 | 0.2 | 0.0498 | 0.192 | 0.261 |
| 90 | 10299.63 | 0.21 | 0.215 | 0.287 | 0.55 |
| 91 | 10319.94 | 0.18 | 0.249 | 0.597 | 0.237 |
| 92 | 11027.52 | 0.77 | < 0.000001 | < 0.000001 | < 0.000001 |
| 93 | 11192.16 | 0.05 | 0.598 | 0.624 | 0.261 |
| 94 | 11305.18 | 0.31 | 0.649 | 0.96 | 0.000745 |
| 95 | 11345.34 | 0.93 | 0.166 | 0.468 | 0.000259 |
| 96 | 11851.7 | 0.11 | 0.564 | 0.736 | < 0.000001 |
| 97 | 11882.45 | 0.24 | 0.107 | 0.141 | 0.343 |
| 98 | 11996 | 0.37 | 0.00000226 | 0.0000868 | 0.0344 |
| 99 | 12039.59 | 0.58 | < 0.000001 | < 0.000001 | 0.0877 |
| 100 | 12213.17 | 0.62 | 0.00000106 | < 0.000001 | 0.000601 |
| 101 | 12343.26 | 0.3 | 0.0167 | 0.0697 | 0.0286 |
| 102 | 12524.2 | 0.18 | 0.105 | 0.914 | 0.0000407 |
| 103 | 12726.71 | 1.98 | 0.0312 | 0.0209 | 0.0356 |
| 104 | 12815.96 | 1 | < 0.000001 | 0.0000017 | 0.606 |
| 105 | 13127.53 | 0.58 | 0.0286 | 0.0299 | < 0.000001 |
| 106 | 13167.02 | 0.43 | 0.000741 | 0.0000884 | < 0.000001 |
| 107 | 13276.82 | 0.06 | 0.598 | 0.708 | 0.0721 |
| 108 | 13433.3 | 0.57 | 0.00000108 | < 0.000001 | 0.0333 |
| 109 | 13539.8 | 1.59 | 0.141 | 0.11 | 0.00309 |
| 110 | 13628.78 | 0.07 | 0.641 | 0.671 | 0.0752 |
| 111 | 14311.3 | 2.08 | 0.00000229 | < 0.000001 | < 0.000001 |
| 112 | 14781.37 | 0.55 | < 0.000001 | < 0.000001 | 0.00000242 |
| 113 | 15197.41 | 0.14 | 0.00622 | 0.017 | 0.78 |
| 114 | 15625.72 | 0.53 | 0.000359 | < 0.000001 | < 0.000001 |
| 115 | 16087.6 | 0.95 | 0.0105 | 0.0000135 | < 0.000001 |
| 116 | 16683.39 | 0.03 | 0.521 | 0.597 | 0.172 |

DAve: Difference between the maxima land the minimal average peak intensity of all snails populations.
PTTA: *P*-value obtained throught-test. PWKW : *P*-value obtained through Wilcoxon/Kruskal-Wallis test. PAD : *P*-value obtained through Anderson-Darling test.


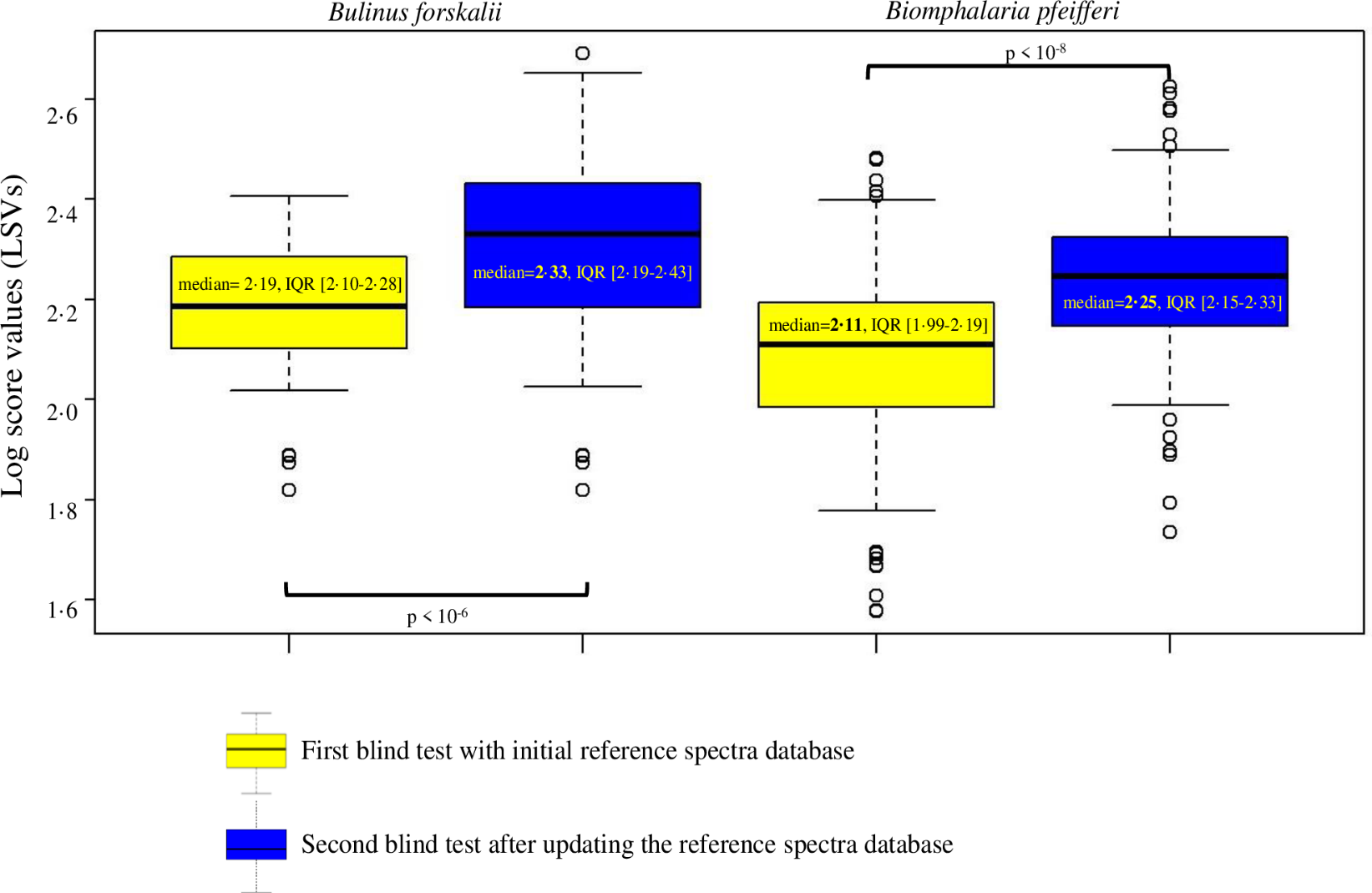


**Fig. S1.** Graphical representation showing the classification of the LSVs of the first and second blind test according to the species of *Biomphalaria pfeifferi* and *Bulinus forskalii*
