# Supplementary material for: Matrix-assisted laser desorption/ionization time-of-flight mass spectrometry traces the geographical source of Biomphalaria pfeifferi and Bulinus forskalii, involved in schistosomiasis transmission
Source: Infect Dis Poverty. 2024 Jan 29;13:11. doi: 10.1186/s40249-023-01168-y (PMC10823745; doi:10.1186/s40249-023-01168-y)
Supplement: Supplementary file 2 — Additional file 2: Representative sequences of Bi. pfeifferi and Bu. forskalii deposited in GenBank. [file 40249_2023_1168_MOESM2_ESM.docx]

**Additional file 2**

**Representative sequences of Biomphalaria pfeifferi and Bulinus forskalii deposited in GenBank**.

> OM535893_COIgene_SRD_S20BFI8_Bulinus_forskalii_Senegal

GTCTAGTAGGAACAGGTCTCTCTTTATTAATTCGACTTGAATTAGGAACAACTTCTGTACTTATAGATGAACATTTTTATAATGTAATTGTTACAGCACATGCTTTTATTATAATTTTTTTTATAGTAATACCTATAATAATTGGTGGATTTGGTAACTGAATGGTTCCTTTATTAATTGGTGCCCCAGATATAAGATTCCCTCGAATAAATAATATATCATTTTGGTTGCTTCCTCCTTCATTTATTTTGTTATTAGTGTCTTCAATAGTTGAAGGAGGGGTTGGAACTGGTTGAACAGTTTACCCTCCTTTAAGAGGGCCAATTGCTCATGGGGGAGCTTCTGTTGATTTAGCTATTTTTTCTCTTCACTTAGCAGGTATATCTTCTATTTTAGGTGCTATTAATTTTATTACTACTATTTTTAATATACGAGCTCCTGGTATTACTATGGAGCGATTATCTTTATTTGTTTGGTCGGTTTTAATTACAGCTTTTTTATTGCTTCTATCTTTACCAGTTTTAGCAGGTGCTATTACTATGCTTTTAACAGATCGTAATTTTAATACTAGATTTTTTGATCCTGCAG

> OM535894_COIgene_SRD_S20BFI10_Bulinus_forskalii_Senegal

GTCTAGTAGGAACAGGTCTCTCTTTATTAATTCGACTTGAATTAGGAACAACTTCTGTACTTATAGATGAACATTTTTATAATGTAATTGTTACAGCACATGCTTTTATTATAATTTTTTTTATAGTAATACCTATAATAATTGGTGGATTTGGTAACTGAATGGTTCCTTTATTAATTGGTGCCCCAGATATAAGATTCCCTCGAATAAATAATATATCATTTTGGTTGCTTCCTCCTTCATTTATTTTGTTATTAGTGTCTTCAATAGTTGAAGGAGGGGTTGGAACTGGTTGAACAGTTTACCCTCCTTTAAGAGGGCCAATTGCTCATGGGGGAGCTTCTGTTGATTTAGCTATTTTTTCTCTTCACTTAGCAGGTATATCTTCTATTTTAGGTGCTATTAATTTTATTACTACTATTTTTAATATACGAGCTCCTGGTATTACTATGGAGCGATTATCTTTATTTGTTTGGTCGGTTTTAATTACAGCTTTTTTATTGCTTCTATCTTTACCAGTTTTAGCAGGTGCTATTACTATGCTTTTAACAGATCGTAATTTTAATACTAGATTTTTTGATCCTGCAG

> OM535895_COIgene_SRD_KABFNI1_Bulinus_forskalii_Senegal

GTCTAGTAGGAACAGGTCTCTCTTTATTAATTCGACTTGAATTAGGAACAACTTCTGTACTTATAGATGAACATTTTTATAATGTAATTGTTACAGCACATGCTTTTATTATAATTTTTTTTATAGTAATACCTATAATAATTGGTGGATTTGGTAACTGAATGGTTCCTTTATTAATTGGTGCCCCAGATATAAGATTCCCTCGAATAAATAATATATCATTTTGGTTGCTTCCTCCTTCATTTATTTTGTTATTAGTGTCTTCAATAGTTGAAGGAGGGGTTGGAACTGGTTGAACAGTTTACCCTCCTTTAAGAGGGCCAATTGCTCATGGGGGAGCTTCTGTTGATTTAGCTATTTTTTCTCTTCACTTAGCAGGTATATCTTCTATTTTAGGTGCTATTAATTTTATTACTACTATTTTTAATATACGAGCTCCTGGTATTACTATGGAGCGATTATCTTTATTTGTTTGGTCGGTTTTAATTACAGCTTTTTTATTGCTTCTATCTTTACCAGTTTTAGCAGGTGCTATTACTATGCTTTTAACAGATCGTAATTTTAATACTAGATTTTTTGATCCTGCAG

> ON077052_COIgene_Diourbel_KHBF2_Bulinus_forskalii_Senegal

CTAGTAGGAACAGGTCTCTCTTTATTAATTCGACTTGAATTAGGAACAACTTCTGTACTTATAGATGAACATTTTTATAATGTAATTGTTACAGCACATGCTTTTATTATAATTTTTTTTATAGTAATACCTATAATAATTGGTGGATTTGGTAACTGAATGGTTCCTTTATTAATTGGTGCCCCAGATATAAGATTCCCTCGAATAAATAATATATCATTTTGGTTGCTTCCTCCTTCATTTATTTTGTTATTAGTGTCTTCAATAGTTGAAGGAGGGGTTGGAACTGGTTGAACAGTTTACCCTCCTTTAAGAGGGCCAATTGCTCATGGGGGAGCTTCTGTTGATTTAGCTATTTTTTCTCTTCATTTAGCAGGTATATCTTCTATTTTAGGTGCTATTAATTTTATTACTACTATTTTTAATATACGAGCTCCTGGTATTACCATGGAGCGATTATCTTTATTTGTTTGGTCAGTTTTAATTACAGCTTTTTTATTGCTTCTATCTTTACCAGTTTTAGCAGGTGCTATTACTATGCTTTTAACAGATCGTAATTTTAATA

> ON077053_COIgene_Diourbel_KHBF3_Bulinus_forskalii_Senegal

CTAGTAGGAACAGGTCTCTCTTTATTAATTCGACTTGAATTAGGAACAACTTCTGTACTTATAGATGAACATTTTTATAATGTAATTGTTACAGCACATGCTTTTATTATAATTTTTTTTATAGTAATACCTATAATAATTGGTGGATTTGGTAACTGAATGGTTCCTTTATTAATTGGTGCCCCAGATATAAGATTCCCTCGAATAAATAATATATCATTTTGGTTGCTTCCTCCTTCATTTATTTTGTTATTAGTGTCTTCAATAGTTGAAGGAGGGGTTGGAACTGGTTGAACAGTTTACCCTCCTTTAAGAGGGCCAATTGCTCATGGGGGAGCTTCTGTTGATTTAGCTATTTTTTCTCTTCATTTAGCAGGTATATCTTCTATTTTAGGTGCTATTAATTTTATTACTACTATTTTTAATATACGAGCTCCTGGTATTACCATGGAGCGATTATCTTTATTTGTTTGGTCAGTTTTAATTACAGCTTTTTTATTGCTTCTATCTTTACCAGTTTTAGCAGGTGCTATTACTATGCTTTTAACAGATCGTAATTTTAATA

> OM535896_COIgene_Kedougou_MP05Bi1_Biomphalaria_pfeifferi_Senegal

CTAGTTGGTACTGGATTATCATTATTAATTCGTTTAGAATTAGGTACTACTCTTGTTTTGATAGATGAACACTTTTATAATGTTATTGTTACAGCTCATGCTTTCATTATAATTTTTTTTATAGTTATACCTATAATAATTGGTGGATTTGGTAATTGAATGATTCCTTTATTAATTGGTGCTCCTGATATAAGTTTTCCTCGAATAAATAATATATCATTTTGATTGCTTCCACCTTCTTTTATTTTATTATTAGTTTCTAGAATAGTTGAAGGTGGGGTAGGAACAGGTTGAACTGTATATCCTCCTTTAAGTGGTCCTATTGCTCATGGAGGTGCTTCTGTAGATTTGGCTATTTTTTCTTTGCATTTAGCAGGTATAAGTTCAATTTTAGGTGCTATTAATTTTATTACTACAATTTTTAATATACGTGCTCCTGGTATTACAATGGAACGTTTATCATTATTTGTTTGATCTGTTCTAGTTACAGCATTTTTACTTTTATTATCTTTGCCTGTTTTAGCTGGGGCTATTACAATATTATTAACTGATCGAAATTTTAATA

> OM535897_COIgene_Kedougou_MP05Bi2_Biomphalaria_pfeifferi_Senegal

CTAGTTGGTACTGGATTATCATTATTAATTCGTTTAGAATTAGGTACTACTCTTGTTTTGATAGATGAACACTTTTATAATGTTATTGTTACAGCTCATGCTTTCATTATAATTTTTTTTATAGTTATACCTATAATAATTGGTGGATTTGGTAATTGAATGATTCCTTTATTAATTGGTGCTCCTGATATAAGTTTTCCTCGAATAAATAATATATCATTTTGATTGCTTCCACCTTCTTTTATTTTATTATTAGTTTCTAGAATAGTTGAAGGTGGGGTAGGAACAGGTTGAACTGTATATCCTCCTTTAAGTGGTCCTATTGCTCATGGAGGTGCTTCTGTAGATTTGGCTATTTTTTCTTTGCATTTAGCAGGTATAAGTTCAATTTTAGGTGCTATTAATTTTATTACTACAATTTTTAATATACGTGCTCCTGGTATTACAATGGAACGTTTATCATTATTTGTTTGATCTGTTCTAGTTACAGCATTTTTACTTTTATTATCTTTGCCTGTTTTAGCTGGGGCTATTACAATATTATTAACTGATCGAAATTTTAATA

> ON062292_16Sgene_SRD_S20BFI8_Bulinus_forskalii_Senegal

CCTATTGAAAAAGTATAATAGGTTAGTTCTGCCCAGTGTAATTTATAAATGGCCGCAGTACCCTGACTGTGCTAAGGTAGCATAATCAATTGGCTTTTAATTGAAGTCTGGAATGAAAGGATTAATGGAGTTTAACTGTCTTACTTGTATAAATTTAAACTTATTTAAAAAGTGAAAATACTTTTTTAAAAATAAAAGACGAGAAGACCCTAAAAGTTTTTAGAATTATTCTTTTTGTTGGGGCGACAATTTAGTAAGAAAACCTATTTATTTAATAAGGCGAATTTTTTAAGTAAAAAAAAACTACTTTAGGGATAACAGCATAATTTTAAAAAGTTTATGACCTCGATGTTGGACTAGGAACTTTATGGCTAGCAGTCAAAATAGATTTATTCTGTTCGAATAATAATATCCT

> ON062293_16Sgene_SRD_S20BFI10_Bulinus_forskalii_Senegal

CCTATTGAAAAAGTATAATAGGTTAGTTCTGCCCAGTGTAATTTATAAATGGCCGCAGTACCCTGACTGTGCTAAGGTAGCATAATCAATTGGCTTTTAATTGAAGTCTGGAATGAAAGGATTAATGGAGTTTAACTGTCTTACTTGTATAAATTTAAACTTATTTAAAAAGTGAAAATACTTTTTTAAAAATAAAAGACGAGAAGACCCTAAAAGTTTTTAGAATTATTCTTTTTGTTGGGGCGACAATTTAGTAAGAAAACCTATTTATTTAATAAGGCGAATTTTTTAAGTAAAAAAAAACTACTTTAGGGATAACAGCATAATTTTAAAAAGTTTATGACCTCGATGTTGGACTAGGAACTTTATGGCTAGCAGTCAAAATAGATTTATTCTGTTCGAATAATAATATCCT

> ON062294_16Sgene_SRD_KABFNI1_Bulinus_forskalii_Senegal

CCTATTGAAAAAGTATAATAGGTTAGTTCTGCCCAGTGTAATTTATAAATGGCCGCAGTACCCTGACTGTGCTAAGGTAGCATAATCAATTGGCTTTTAATTGAAGTCTGGAATGAAAGGATTAATGGAGTTTAACTGTCTTACTTGTATAAATTTAAACTTATTTAAAAAGTGAAAATACTTTTTTAAAAATAAAAGACGAGAAGACCCTAAAAGTTTTTAGAATTATTCTTTTTGTTGGGGCGACAATTTAGTAAGAAAACCTATTTATTTAATAAGGCGAATTTTTTAAGTAAAAAAAAACTACTTTAGGGATAACAGCATAATTTTAAAAAGTTTATGACCTCGATGTTGGACTAGGAACTTTATGGCTAGCAGTCAAAATAGATTTATTCTGTTCGAATAATAATATCCT

> ON062295_16Sgene_SRD_KABFNI5_Bulinus_forskalii_Senegal

CCTATTGAAAAAGTATAATAGGTTAGTTCTGCCCAGTGTAATTTATAAATGGCCGCAGTACCCTGACTGTGCTAAGGTAGCATAATCAATTGGCTTTTAATTGAAGTCTGGAATGAAAGGATTAATGGAGTTTAACTGTCTTACTTGTATAAATTTAAACTTATTTAAAAAGTGAAAATACTTTTTTAAAAATAAAAGACGAGAAGACCCTAAAAGTTTTTAGAATTATTCTTTTTGTTGGGGCGACAATTTAGTAAGAAAACCTATTTATTTAATAAGGCGAATTTTTTAAGTAAAAAAAAAACTACTTTAGGGATAACAGCATAATTTTAAAAAGTTTATGACCTCGATGTTGGACTAGGAACTTTATGGCTAGCAGTCAAAATAGATTTATTCTGTTCGAATAATAATATCCT

> ON062296_16Sgene_Diourbel_KHBF2_Bulinus_forskalii_Senegal

CCTATTGAAAAAGTATAATAGGTTAGTTCTGCCCAGTGTAATTTATAAATGGCCGCAGTACCCTGACTGTGCTAAGGTAGCATAATCAATTGGCTTTTAATTGAAGTCTGGAATGAAAGGATTAATGGAGTTTAACTGTCTTACTTGTATAAATTTAAACTTATTTAAAAAGTGAAAATACTTTTTTAAAAATAAAAGACGAGAAGACCCTAAAAGTTTTTAGAATTATTCTTTTTGTTGGGGCGACAATTTAGTAAGAAAACCTATTTATTTAATAAGGCGAATTTTTTAAGTAAAAAAAAACTACTTTAGGGATAACAGCATAATTTTAAAAAGTTTATGACCTCGATGTTGGACTAGGAACTTTATGGCTAGCAGTCAAAATAGATTTATTCTGTTCGAATAATACTATCCT

> ON062297_16Sgene_Diourbel_KHBF3_Bulinus_forskalii_Senegal

CCTATTGAAAAAGTATAATAGGTTAGTTCTGCCCAGTGTAATTTATAAATGGCCGCAGTACCCTGACTGTGCTAAGGTAGCATAATCAATTGGCTTTTAATTGAAGTCTGGAATGAAAGGATTAATGGAGTTTAACTGTCTTACTTGTATAAATTTAAACTTATTTAAAAAGTGAAAATACTTTTTTAAAAATAAAAGACGAGAAGACCCTAAAAGTTTTTAGAATTATTCTTTTTGTTGGGGCGACAATTTAGTAAGAAAACCTATTTATTTAATAAGGCGAATTTTTTAAGTAAAAAAAAACTACTTTAGGGATAACAGCATAATTTTAAAAAGTTTATGACCTCGATGTTGGACTAGGAACTTTATGGCTAGCAGTCAAAATAGATTTATTCTGTTCGAATAATACTATCCT
